# Enantiospecific Syntheses of Congested Atropisomers through Chiral Bis(aryne) Synthetic Equivalents

**DOI:** 10.1002/chem.202202473

**Published:** 2022-09-29

**Authors:** Guillaume Dauvergne, Jean‐Valère Naubron, Michel Giorgi, Xavier Bugaut, Jean Rodriguez, Yannick Carissan, Yoann Coquerel

**Affiliations:** ^1^ Aix Marseille Univ CNRS Centrale Marseille, iSm2 Marseille France; ^2^ Aix Marseille Univ CNRS Centrale Marseille FSCM Marseille France

**Keywords:** arynes, atropisomerism, chirality, enantiospecific reactions, polycyclic aromatic hydrocarbons

## Abstract

The synthetic equivalents of the enantiopure binaphthyl bis(aryne) atropisomers derived from BINOL (1,1′‐bi‐2,2′‐naphtol) featuring a stereogenic axis vicinal to the two reactive triple bonds can be generated for the first time in solution in an enantiospecific manner. Using a two‐step sequence based on the bidirectional [4+2] cycloaddition of furan derivatives followed by an aromatizative deoxygenation reaction, several 9,9’‐bianthracenyl‐based atropisomers could be prepared enantiospecifically in high enantiomeric purity. Alternatively, bidirectional reactions with anthracene, 2‐bromostyrene, and perylene as the arynophiles afforded very congested bis(benzotriptycene), bis(tetraphene) and bis(anthra[1,2,3,4‐*ghi*]perylene) nanocarbon atropisomers in equally high enantiomeric purity. In complement, cross reactions with two different arynophiles revealed possible. The unusual atropisomer prototypes described in this study open the way to enantiopure nanographene atropisomers designed for functions.

## Graphical abstract

**The third dimension of arynes**. Synthetic equivalents of enantiopure bis(aryne) atropisomers derived from BINOL can be generated and reacted in solution with various arynophiles for the fully enantiospecific syntheses of congested large polycyclic aromatic hydrocarbon atropisomers.

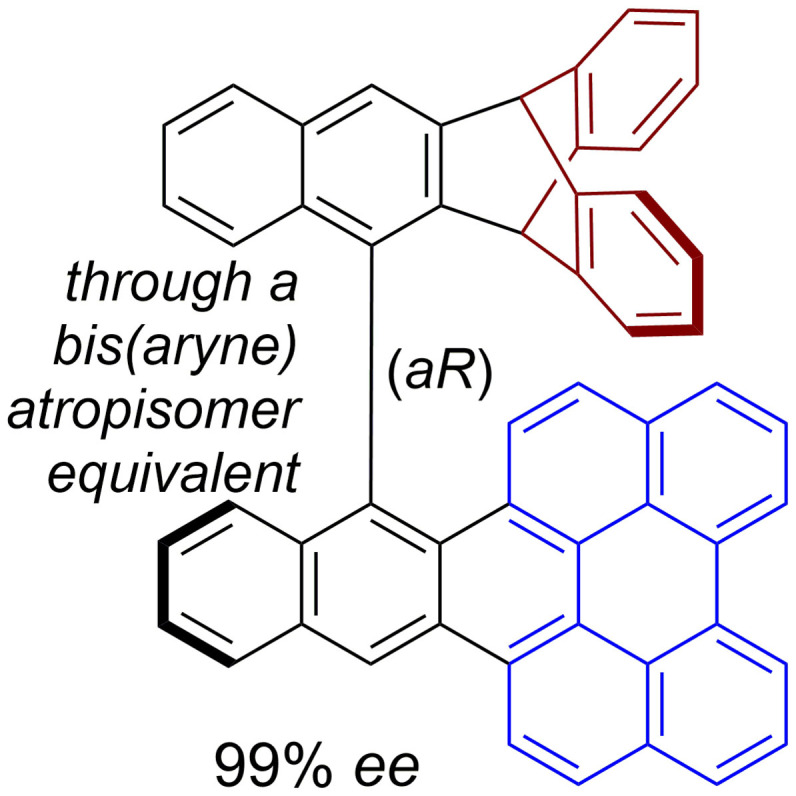

## Introduction

Chirality is a fascinating concept of chemistry, from living organisms to synthetic nanomaterials.^[1]^ Nowadays, the control of chirality at the molecular scale is crucial to the development of drugs and technological applications.^[2]^ Axial chirality and atropisomerism can result from the restricted rotation around a single bound in molecules, for instance in hindered biphenyl derivatives. In these molecules, the two enantiomers, also called atropisomers, are conformers, and their interconversion occurs by rotation around the C(*sp* ^*2*^)−C(*sp* ^*2*^) single bond. Providing the energy barrier to rotation around this bond is high enough, atropisomers can be obtained as enantiomerically pure compounds, either by the resolution of racemic material or by enantioselective catalytic approaches.^[3]^ While the importance of chirality in drugs was realized decades ago, it is only recently that atropisomers have gained popularity in medicinal chemistry.^[4]^ Additionally, atropisomers are important in enantioselective catalysis, either as ligands for transition metals or as organocatalysts, and they play a key role in the field of functional molecules such as molecular motors.^[3]^ Chirality in polycyclic aromatic hydrocarbons (PAH) has gained considerable momentum in chemical sciences because its existence raises new questions, and its control offers new opportunities. With the progress of organic synthesis over the past decade, some incredibly beautiful large chiral PAH nanographenes and multihelicenes with amazing properties have been prepared.^[5]^ The synthesis of large PAH atropisomers have received much less attention, and the development of a general approach for their synthesis is desirable.

*ortho*‐Arynes, or simply arynes, are now firmly established as useful synthetic tools in organic chemistry with a variety of applications for the synthesis of partially or fully aromatic molecules, from complex natural products to large polycyclic aromatic hydrocarbons (PAH).^[6]^ Arynes generally react with nucleophiles in addition reactions, and with unsaturated molecules in cycloaddition reactions. For example, they have been employed for the synthesis of biaryl atropisomers through the formation of the C(*sp* ^*2*^)−C(*sp* ^*2*^) stereogenic single bond,^[7]^ and some achiral and racemic biphenyl‐based arynes have been described.^[8]^ Because of their unique electronic structure,^[9]^ arynes are short‐lived species that must be generated in situ, most commonly through the *ortho*‐elimination of a suitable aromatic precursor.^[6d]^ Synthetic applications of mono(arynes) having a single ‘triple bond’ – an oversimplification of their electronic structure – are legion. Some synthetic equivalents of monocyclic bis(arynes) having two ‘triple bonds’ embedded in the same ring have been described.^[10]^ Bicyclic bis(arynes)^[11]^ and polycyclic tris(arynes)^[12]^ synthetic equivalents have also been described recently for the construction of planar or achiral large PAH. The possibility for having several reactive aryne functionalities present on the same molecule – successively not simultaneously as discussed below – has further expanded the synthetic options available and allows for the rapid and efficient construction of complex aromatic molecules.

**Scheme 1:**
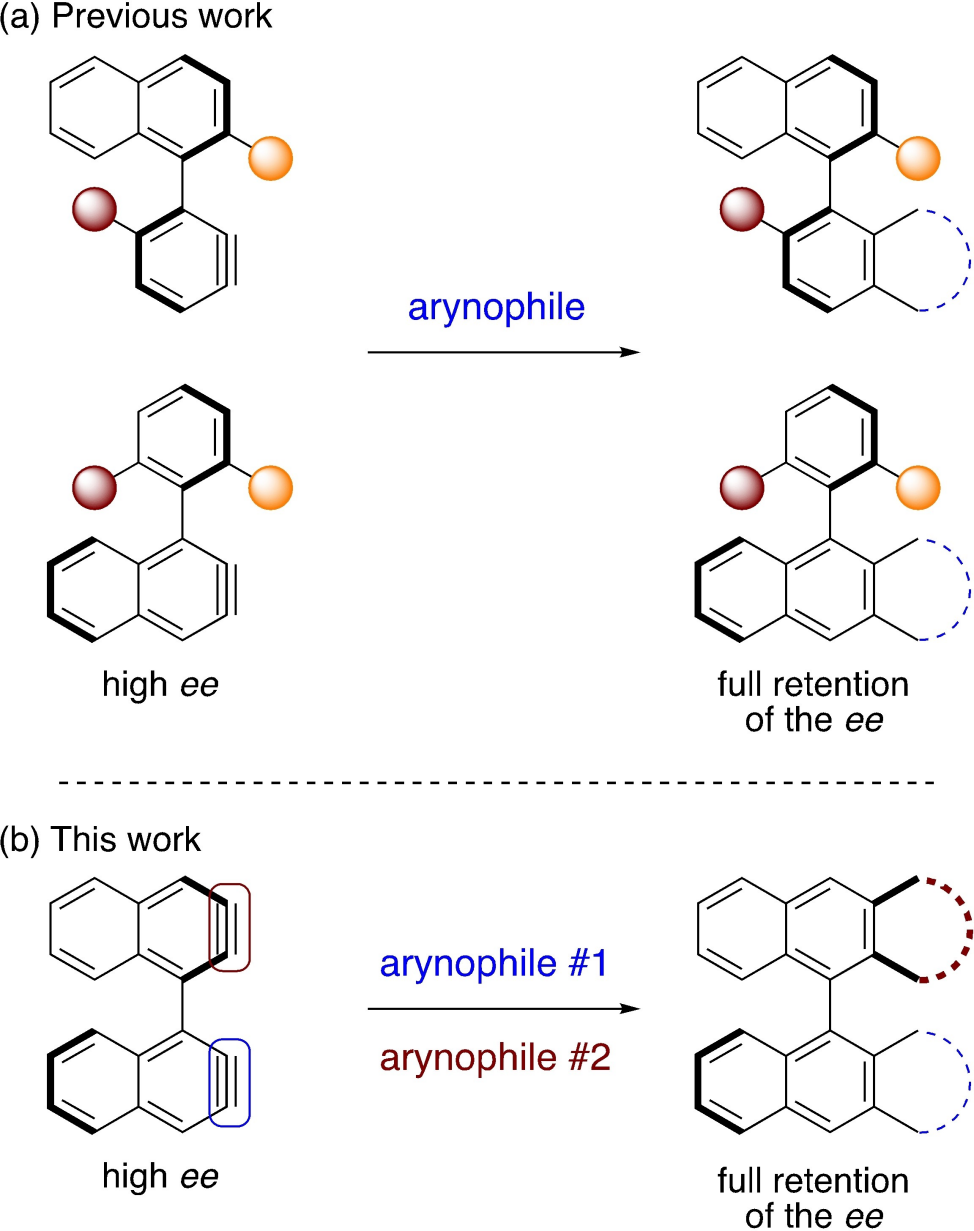
An overview of aryne atropisomers. (a) Aryne atropisomers with a 1‐phenylnaphthalene backbone.^[13]^ (b) A formal bis(aryne) atropisomer derived from (*R*)‐BINOL.

Recently, the enantiospecific generation and trapping reactions of nonracemic mono(aryne) atropisomers based on a 1‐phenylnaphthalene backbone were described (Scheme 1a), which unlocked new options for the stereocontrolled synthesis of nonracemic biaryl atropisomers.^[13]^ Herein, the enantiospecific syntheses of large and congested bianthracenyl‐based PAH atropisomers are described based on the reactivity of enantiopure bis(aryne) atropisomer synthetic equivalents derived from BINOL (Scheme 1b). The atropisomers prototypes prepared this way were obtained with *ee*>=98 %, and present original chirality patterns that would be very difficult if not impossible to reach with comparable efficiency by other methods, even in their racemic form.

## Results and discussion

It was hypothesized that the bis(aryne) atropisomer shown in Scheme 1b could formally be derived from a suitable precursor obtained from BINOL (1,1′‐bi‐2,2′‐naphtol). Early on, the bis(*ortho*‐trimethylsilylnaphthyl triflate) Kobayashi‐type precursor derived from BINOL was considered, but was rapidly abandoned because of its propensity to undergo competitive two‐fold thia‐Fries rearrangement^[14]^ instead of the expected fluoride‐induced *ortho*‐elimination (see Supporting Information for details, CCDC 2173780, Figure S1, Table S1, Scheme S1). Instead, it was found that both atropisomers of the bis(*ortho*‐iodonaphthyl triflate) Suzuki‐type precursor **1** treated with excess trimethylsilylmethylmagnesium chloride can serve as a source of the corresponding bis(aryne) atropisomers, in fact their synthetic equivalents. Precursors (*aS*)‐**1** and (*aR*)‐**1** were obtained quantitatively in 99 % *ee* by a two‐fold triflation of the corresponding enantiopure 3,3’‐diiodo‐1,1’‐binaphthyl‐2,2’‐diol atropisomers derived from BINOL^[15]^ (4 steps from BINOL, 77 %, see the Supporting Information for the syntheses details).

One popular approach for the annulative π extension (APEX)^[16]^ of arynes is to take advantage of their generally efficient [4+2] cycloadditions with furan derivatives followed by some aromatizative deoxygenation reaction.^[6]^ Using this two‐step approach, the bidirectional reaction between precursor (*aS*)‐**1** (99 % *ee*) and 2,5‐dimethylfuran furnished the original 1,1′,4,4′‐tetramethyl‐9,9’‐bianthracene (*aS*)‐**2** (57 % over two steps, 99 % *ee*), and the enantiomeric atropisomer (*aR*)‐**2** (not shown) was obtained in similar yield and enantiomeric purity from the bis(aryne) atropisomer precursor (*aR*)‐**1**. The nature and homogeneity of atropisomer (*aS*)‐**2** was deduced from its spectroscopic data, its *ee* was determined by analytical HPLC on a chiral stationary phase, and its absolute configuration was confirmed by electronic circular dichroism (ECD) spectroscopy (see Supporting Information, Figure S2). A similar reaction sequence with 3,4‐dibromofuran furnished 2,2′,3,3′‐tetrabromo‐9,9’‐bianthracene (*aS*)‐**3** (52 % over two steps, 98 % *ee*). The structure and the absolute configuration of (*aS*)‐**3** were now confirmed by X‐ray diffraction analysis of a monocrystal (see Supporting Information, CCDC 2173781, Figure S3, Table S2). In another set of experiments, the bis(aryne) atropisomer precursors (*aS*)‐**1** and (*aR*)‐**1** were engaged in [4+2] cycloadditions with representative π‐conjugated aromatic hydrocarbons, especially anthracene, 2‐bromostyrene, and perylene. The reaction with anthracene furnished the bis(benzotriptycene) atropisomer (*aS*)‐**4** (57 %, 99 % *ee*), the structure and absolute configuration of which were confirmed by X‐ray diffraction analysis of a monocrystal (see Supporting Information, CCDC 2173779, Figure S4, Table S3). Chiral triptycene derivatives are emerging for applications in functional supramolecular materials.^[17]^ The original bis(benzotriptycene) atropisomer (*aS*)‐**4** exhibits a unique chirality figure with the position and orientation of six distinct π systems controlled in space. The reaction with 2‐bromostyrene is bringing some degree of complexity to the synthesis because it calls for a two‐fold regioselective transformation forming the most hindered regioisomer. It was previously discovered that 2‐bromostyrene reacts with arynes in regioselective [4+2] cycloadditions followed by spontaneous aromatizative dehydrobromimation.^[18]^ Its reaction with the bis(aryne) atropisomer precursor (*aR*)‐**1** afforded a mixture of products, the major component of which was unambiguously identified as the expected atropisomer (*aR*)‐**5** (37 %, 99 % *ee*), probing good regioselectivity of the overall transformation despite the severely crowded nature of atropisomer (*aR*)‐**5** around the stereogenic axis. Finally, the two‐fold reaction with perylene revealed challenging; nevertheless, the nanographene atropisomer (*aR*)‐**6** (4 %, 98 % *ee*) could be isolated. This atropisomer is extremely congested around the stereogenic axis, which justifies, in part, the low yield of its synthesis.

**Figure 1:**
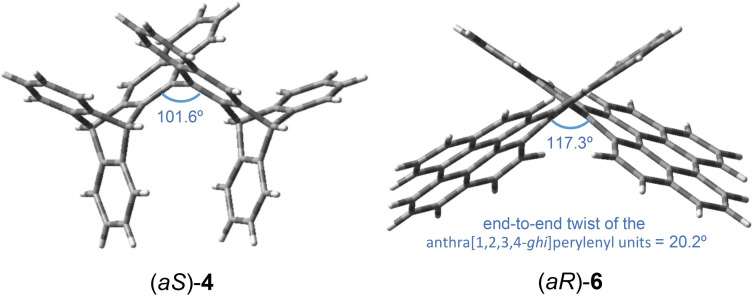
3D representations of (*aS*)‐**4** (left) and (*aR*)‐**6** (right) obtained by single crystal X‐ray diffraction analysis and DFT modelling (M062X‐D3/cc‐pVDZ), respectively.

Congestion around the stereogenic axis in the bianthracenyl‐based atropisomers can be assessed by measuring the torsional angle between the two largest *ortho‐*substituents on either side of the stereogenic axis. For instance, torsion was thus measured at 92.2° in undistorted (*aS*)‐**3** from structural data obtained by single crystal X‐ray analysis. In (*aS*)‐**4**, torsion was measured at 101.6° also using structural data obtained by single crystal X‐ray analysis (Figure 1, left). Measurements of torsion in (*aR*)‐**5** and (*aR*)‐**6**, now using computational modeling (DFT, M062X‐D3/cc‐pVDZ) to obtain the geometries, revealed torsional angles of 106.4° and 117.3°, respectively. In the two latter cases, the compactness of the molecules forces the tetraphenyl and anthra[1,2,3,4‐*ghi*]perylenyl units to adopt a (*P*)‐configured helical conformation with end‐to‐end twist angles of 15.3° and 20.2°, respectively [see Figure 1, right, for (*aR*)‐**6**], like in twistacenes.[^10e^, ^19^] Molecule (*aR*)‐**6** exhibits three configurationally stable stereogenic elements, a primary stereogenic axis and two secondary helical twists, and should be regarded as the first example of a bis(twistacene) atropisomer, a new class of chiral PAH with multiple stereogenicities.

**Scheme 2:**
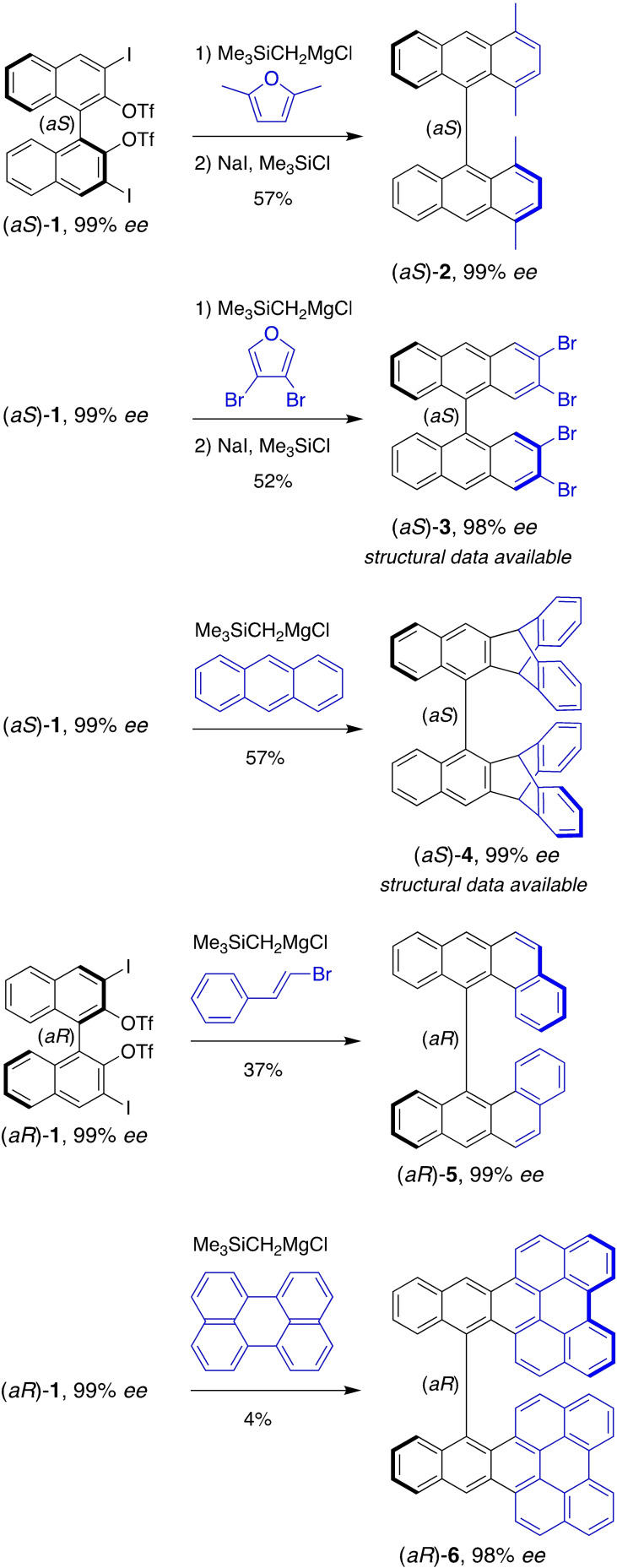
Enantiospecific bidirectional cycloadditions of the bis(aryne) atropisomer synthetic equivalent derived from precursors (*aS*)‐**1** and (*aR*)‐**1**. Reactions were generally performed with 2 equiv. of the arynophile and 10 equiv. of Me_3_SiCH_2_MgCl for 16 h in Et_2_O at 0 °C. Yields were determined for isolated material, and the enantiomer ratios were determined by analytical HPLC on chiral stationary phases.

**Scheme 3:**
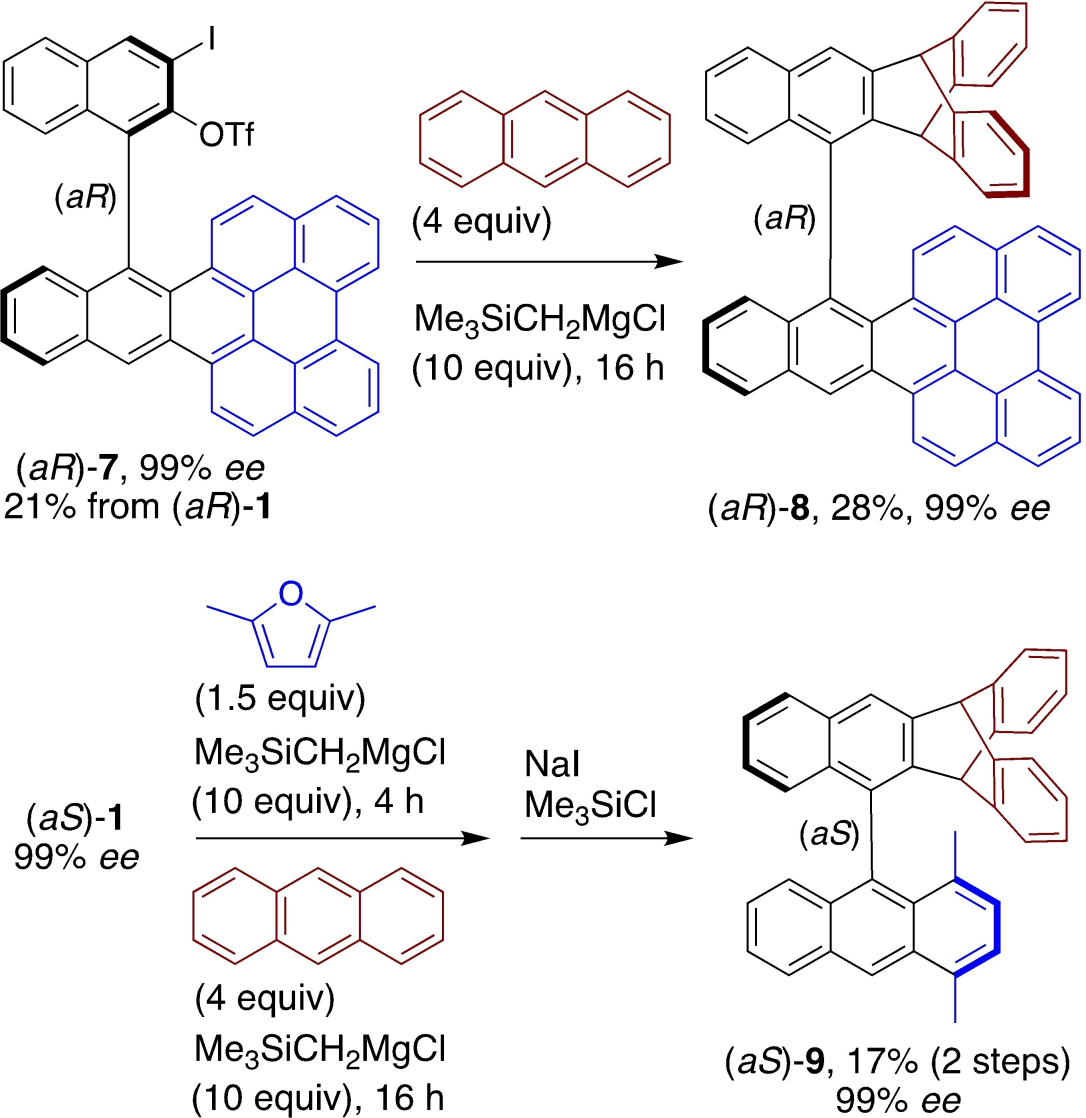
Enantiospecific cycloadditions of the bis(aryne) atropisomer synthetic equivalent with different arynophiles. Yields were determined for isolated material, and the enantiomer ratios were determined by analytical HPLC on chiral stationary phases.

The reaction of the bis(aryne) atropisomer precursor (*aR*)‐**1** with perylene shown in Scheme 2 can be interrupted (2 h reaction time instead of 16 h) to afford predominantly the mono(adduct) (*aR*)‐**7** (21 %, 99 % *ee*) having one perylene unit and one preserved *ortho*‐iodonaphthyl triflate unit (Scheme 3). Using (*aR*)‐**7** as the precursor of an aryne atropisomer in a reaction with anthracene, it was possible to obtain the cross biaryl atropisomer (*aR*)‐**8** (28 %, 99 % *ee*). The possibility for incorporating two different arynophiles in a ‘one‐pot’ process was also demonstrated: successive treatment of (*aS*)‐**1** with trimethylsilylmethylmagnesium chloride, 2,5‐dimethylfuran, trimethylsilylmethylmagnesium chloride again, and anthracene afforded a mixture of diastereomeric oxa‐bridged compounds, the aromatizative deoxygenation of which with NaI/Me_3_SiCl afforded atropisomer (*aS*)‐**9** (17 % over the two steps, 99 % *ee*). The original triptycene^[17]^ atropisomers (*aR*)‐**8** and (*aS*)‐**9** obtained in 99 % *ee* exhibit four distinct π systems, including a relatively large π‐extended one, with controlled positions and orientations in space.

For applications, it is important to evaluate the enantiomerization barriers of the atropisomers **2–6**, **8**, and **9** and their congeners. They should all be comparable to or greater than the rotation barrier around the C(*sp* ^*2*^)−C(*sp* ^*2*^) single bond of achiral 9,9’‐bianthracene. Using the SMD(diethyl ether)/M062X‐D3/aug‐cc‐pVDZ//M062X‐D3/cc‐pVDZ level of theory, the rotation barrier for 9,9’‐bianthracene was computed as a two‐step process via a high‐energy anti‐folded *C*_*2 h*_ symmetric intermediate and Δ*G* ^≠^_rot_=189.6 kJ mol^−1^ at 25 °C (See the Supporting Information, Figure S7). Thus, all atropisomers herein and their congeners can be regarded as configurationally stable for applications at temperatures up to 180 °C with t_1/2_(racemization) >8 years at this temperature.

Calculation of the barrier to enantiomerization of the elusive bis(aryne) atropisomer shown in Scheme 1b by a similar DFT method gave Δ*G* ^≠^_enant_=5.4 kJ mol^−1^ (see Supporting Information, Figure S5), indicating that if it is generated at 0 °C it will racemize in a few picoseconds. This results from the absence of two H atoms *ortho* to the stereogenic axis causing no van der Waals interactions to hamper the rotation around the C(*sp* ^*2*^)−C(*sp* ^*2*^) stereogenic single bond in the planar transition state corresponding to enantiomerization. The reaction half‐life of arynes in solution can be evaluated in the magnitude of tens to a few hundred nanoseconds at 0 °C.^[13b]^ The enantiomerization rate of the elusive bis(aryne) atropisomer derived from BINOL is thus more than 10^6^ times greater than its reaction rate, indicating that if it was generated in the reactions described herein, only racemic products would have been obtained. However, in all cases, a complete enantiospecificity was observed experimentally, demonstrating quantitatively that at no time the bis(aryne) atropisomer is generated, and that two aryne atropisomers are successively generated instead. This is also likely the case for all previously described bis‐ and tris(arynes).[^10^, ^11^, ^12^] The barrier to enantiomerization of the mono(aryne) generated from precursors (*aS*)‐**1** and (*aR*)‐**1** was computed at 109.5 kJ mol^−1^ (see Supporting Information, Figure S6), a value compatible with the observed complete enantiospecificity.

Deposition Numbers 2173779 (for (*aS*)‐**4**), 2173780 (for (*aR*)‐**A**), and 2173781 (for (*aS*)‐**3**) contain the supplementary crystallographic data for this paper. These data are provided free of charge by the joint Cambridge Crystallographic Data Centre and Fachinformationszentrum Karlsruhe Access Structures service.

## Conclusions

Nonracemic bis(aryne) atropisomers synthetic equivalents could be generated enantiospecifically from their so‐called Suzuki‐type precursors derived from BINOL having two *ortho*‐iodoaryl triflate moieties prone to slow *ortho*‐elimination in the presence of excess trimethylsilylmethylmagnesium chloride. These reaction conditions should generally resolve the recurring issue of kinetic competition between thia‐Fries rearrangement and 1,2‐elimination in reactions of hindered *ortho*‐iodoaryl triflate based aryne precursors. Enantiospecific bidirectional cycloadditions with two identical or two different arynophiles could be performed, which resulted in the spatially controlled syntheses of various congested large atropisomers in high enantiomeric purity. The syntheses of anthracene‐, benzotriptycene‐, tetraphene‐, and anthra[1,2,3,4‐*ghi*]perylene‐based prototypical atropisomers are reported through this approach, which appears general. Several of the molecules described herein exhibit an unprecedented chirality pattern due to the unusual congestion around the stereogenic axis. Based on kinetic considerations, it was quantitatively demonstrated that the bis(aryne) atropisomers derived from the enantiomers of BINOL do not exist as chemical species, only the corresponding mono(aryne) atropisomers can be successively generated in solution. The exploration of the chemical, structural, conformational, electronic, photophysical, and chiroptical properties of some of the biaryl atropisomers described herein, their derivatives, and their analogues is ongoing.

## Conflict of interest

The authors declare no conflict of interest.

1

## Supporting information

As a service to our authors and readers, this journal provides supporting information supplied by the authors. Such materials are peer reviewed and may be re‐organized for online delivery, but are not copy‐edited or typeset. Technical support issues arising from supporting information (other than missing files) should be addressed to the authors.

Supporting Information

Supporting Information

Supporting Information

Supporting Information

Supporting Information

Supporting Information

Supporting Information

Supporting Information

Supporting Information

Supporting Information

Supporting Information

Supporting Information

Supporting Information

Supporting Information

## Data Availability

The data that support the findings of this study are available in the supplementary material of this article.
