# Supplementary material for: Enantiospecific Syntheses of Congested Atropisomers through Chiral Bis(aryne) Synthetic Equivalents
Source: Chemistry. 2022 Sep 29;28(68):e202202473. doi: 10.1002/chem.202202473 (PMC10087792; doi:10.1002/chem.202202473)
Supplement: Supplementary file 8 — Supporting Information [file CHEM-28-0-s011.pdf]

Chemistry–A European Journal

Supporting Information

Enantiospecific Syntheses of Congested Atropisomers through Chiral Bis(aryne) Synthetic Equivalents

Guillaume Dauvergne, Jean-Valère Naubron, Michel Giorgi, Xavier Bugaut, Jean Rodriguez, Yannick Carissan,* and Yoann Coquerel*

Enantiospecific Syntheses of Congested Atropisomers through Chiral Bis(aryne) Synthetic Equivalents

Guillaume Dauvergne,[a] Jean-Valère Naubron,[b] Michel Giorgi,[b] Xavier Bugaut,[a] Jean Rodriguez,[a] Yannick Carissan,*[a] Yoann Coquerel*[a]

[a]Aix Marseille Univ, CNRS, Centrale Marseille, iSm2, Marseille, France.
[b]Aix Marseille Univ, CNRS, Centrale Marseille, FSCM, Marseille, France.

Corresponding authors contact information:
yannick.carissan@univ-amu.fr
yoann.coquerel@univ-amu.fr

Supporting Information

Table of Content

1. Synthetic procedures and characterization data S1
2. Computational studies S40

1. Synthetic procedures and characterization data

General information

Reactions were generally carried out under an argon atmosphere in oven-dried reaction vessels in anhydrous solvents. All reagents were weighed and handled in air at room temperature unless otherwise mentioned, and all commercially available reagents were used as received unless otherwise mentioned. Anhydrous dichloromethane, diethyl ether, and toluene were dried by filtration over solid dehydrating agents using a commercial solvent purification system. Anhydrous acetonitrile was obtained directly from commercial sources. Thin layer chromatography was carried out on Merck Kieselgel 60 F_{254} 0.2 mm plates. Visualization was accomplished using ultraviolet light (254 and/or 365 nm) and/or chemical staining with an ethanolic solution of *para*-anisaldehyde with sulphuric acid as appropriate. Purifications were routinely performed using flash chromatography columns packed with 40-63 µm silica gel generally eluted with a mixture pentane/ethyl acetate or pentane/diethyl ether.

Melting points were recorded using Büchi Melting Point B-540 or B-545 apparatus.

NMR data were generally recorded at 298 ± 3 K in deuterated chloroform at 400 MHz or 500 MHz using as internal standards the residual chloroform signal for ^{1}H NMR (δ = 7.26 ppm) and the deuterated solvent signal for ^{13}C NMR (δ = 77.16 ppm). Chemical shifts (δ) are in ppm, coupling constants (*J*) are in Hertz (Hz) and the classical abbreviations are used to describe the signal multiplicities. ^{13}C DEPT135 experiments were systematically conducted to support assignments.

High resolution mass spectra (HRMS) were recorded in triplicate at the Spectropole (http://fr-chimie.univ-amu.fr/spectropole//) on a Waters Synapt G2 HDMS apparatus using a positive electrospray (ESI) ionization source.

Optical Rotations were measured in $CHCl_3$ on an Anton Paar MCP 200 or on an Anton Paar MCP 100 Polarimeter using a sodium lamp (λ 589 nm, D-line). $[\alpha]_D$ values are reported at a given temperature (°C) in degree·cm^{2}·g^{-1} with concentration in g/100mL.

HPLC analyses for the determination of enantiomeric excess were performed on a Merck-Hitachi system equipped with Chiralcel OD3, Chiralpak IA, Chiralpak IB, Chiralpak IB N-5, Chiralpak IE, and Chiralpak IJ analytical columns.

UV-vis and electronic circular dichroism (ECD) spectra were measured on a JASCO J-815 spectrometer equipped with a JASCO Peltier cell holder PTC-423 to maintain the temperature at 20.0 °C. A quartz photoelastic modulator set at l/4 retardation was used to modulate the handedness of the circular polarized light at 50 kHz. A quartz cell of 1 mm of optical path length was used.

Single crystal X-ray diffraction analysis were performed on a Rigaku Oxford Diffraction SuperNova diffractometer. Data collection reduction and multiscan ABSPACK correction were

performed with CrysAlisPro (Rigaku Oxford Diffraction). Using Olex2[1] the structures were solved by intrinsic phasing methods with SHELXT[2] and SHELXL[3] was used for full matrix least square refinement.

The commercial solution of trimethylsilylmethylmagnesium chloride was titrated by reaction with benzaldehyde in diethyl ether (1.0 M) under an argon atmosphere at 23–25 °C, and its concentration determined by ^{1}H NMR by analysis of the crude reaction mixture.

All reactions with nonracemic substrates were optimized with racemic substrates, which furnished the racemic products that were necessary for the determination of suitable conditions for their resolution by analytical HPLC on chiral stationary phases.

Procedures and characterization data for the reactions of the bis(aryne) atropisomer synthetic equivalent

Enantiopure 3,3′-diiodo-1,1′-binaphthyl-2,2′-diols[4] (precursors of (*aS*)-**1** and (*aR*)-**1**) and 3,3′-bistrimethylsilyl-1,1′-binaphthyl-2,2′-diol[5] (precursor of (*aR*)-**A**) were synthesized by known methods. The NaI/Me_3SiCl system for the aromatizative deoxygenation of the oxa-bridged intermediate cycloadducts was identified by Jung and Korrida,[6] and we found these reactions more efficient in dichloromethane than in acetonitrile as reported.

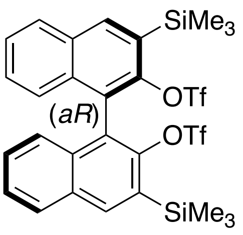

(*aR*)-**A**
Chemical Formula: $C_{28}H_{28}F_6O_6S_2Si_2$
Molecular Weight: 694,81

To a solution of enantiopure (*aR*)-3,3′-bistrimethylsilyl-1,1′-binaphthyl-2,2′-diol (245 mg, 0.57 mmol, >99% ee) in anhydrous diethyl ether (10 mL) was added NaH (60% dispersion in oil, 114 mg, 2.90 mmol) at 0 °C. The resulting mixture was stirred at this temperature for 1 h and Tf_2O (0.49 mL, 2.90 mmol) was added dropwise over 10 min. The reaction was then monitored by TLC until the starting material is no longer detectable (7 days) whereupon a saturated aqueous NH_4Cl solution was added at 0 °C. The resulting mixture was extracted three times

[1] O. V. Dolomanov, L. J. Bourhis, R. J. Gildea, J. A. K. Howard, H. Puschmann, *J. Appl. Cryst.* **2009**, *42*, 339–341.
[2] G. M. Sheldrick, *Acta Cryst.* **2015**, *A71*, 3–8.
[3] G. M. Sheldrick, *Acta Cryst.* **2015**, *C71*, 3–8.
[4] J. Hua, W. Lin, *Org. Lett.* **2004**, *6*, 861–864.
[5] M. Kauch, V. Snieckus, D. Hoppe, *J. Org. Chem.* **2005**, *70*, 7149–7158.
[6] K. Y. Jung, M. Koreeda, *J. Org. Chem.* **1989**, *54*, 5667–5675.

with EtOAc. The combined organic layers were dried over anhydrous Na_2SO_4, filtered, and concentrated under reduced pressure to give the crude product. Purification by flash chromatography on silica gel eluted with pentane/EtOAc = 50:1 afforded (*aR*)-**A** (67 mg, 17%) as an orange solid. **Mp** 169–171 °C ($CDCl_3$); **TLC** (pentane/EtOAc = 50:1) R*f* = 0.86; **HRMS** (ESI+) *m/z* calcd for $C_{28}H_{32}NO_6F_6Si_2S_2$ $[M+NH_4]^+$ 712.1108, found 712.1106; **^{1}H NMR** (400 MHz, $CDCl_3$) δ 8.26 (s, 2H), 7.96 (d, *J* = 8.2 Hz, 2H), 7.55 (ddd, *J* = 8.9, 7.0, 1.1 Hz, 2H), 7.33 (ddd, *J* = 9.1, 7.3, 1.2 Hz, 2H), 7.23 (dd, *J* = 8.5, 0.9 Hz, 2H) ; **^{13}C{^{1}H} NMR** (101 MHz, $CDCl_3$) δ 148.1 (2C), 140.1 (2CH), 134.8 (2C), 133.2 (2C), 132.3 (2C), 128.2 (2CH), 128.1 (2CH), 127.7 (2CH), 127.2 (2CH), 124.8 (2C), 117.8 (q, $^1J_{C-F}$ = 323.0 Hz, $2CF_3$), 0.43 ($6CH_3$); **^{19}F NMR** (376 MHz, $CDCl_3$) δ -74.4. The *ee* of (*aR*)-**A** was not confirmed by HPLC analysis because (*aR*)-**A** was found not suitable for the generation of the corresponding bis(aryne) atropisomer equivalent (see below). The structure and absolute configuration of (*aR*)-**A** were confirmed by X-ray diffraction analysis of a monocrystal (needles) obtained by slow evaporation of chloroform (Figure S1, Table S1, CCDC 2173780).

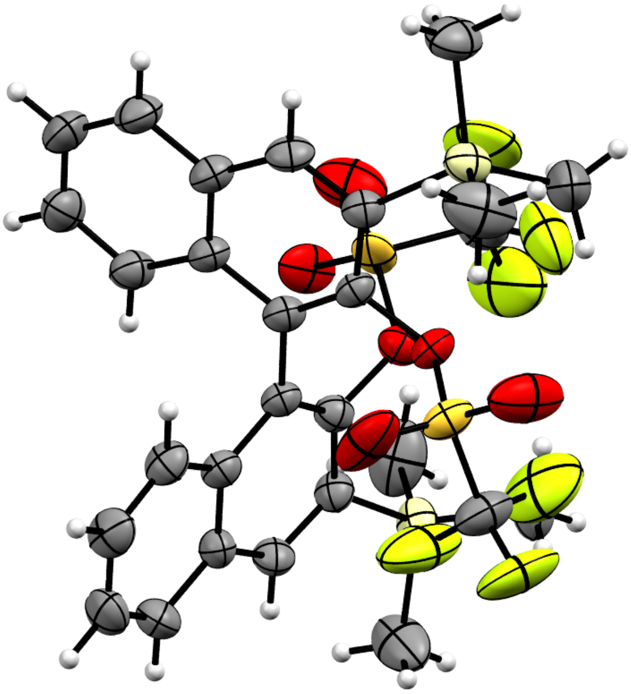

Figure S1. ORTEP representation of (*aR*)-**A** obtained by single-crystal X-ray diffraction analysis. The ellipsoids are drawn at the 50% probability level and H atoms are represented as fixed-size spheres of 0.15 Å radius. These measurements allowed the confirmation of the absolute configuration in (*aR*)-**A**.

Table S1. Crystal data and structure refinement for (*aR*)-**A**.

Empirical formula	$C_{28}H_{28}F_6O_6S_2Si_2$
Formula weight	694.8
Temperature/K	223
Crystal system	orthorhombic
Space group	$P2_12_12_1$
a/Å	10.46854(5)
b/Å	17.46535(10)
c/Å	17.73981(8)
Volume/Å^3	3243.49(3)
Z	4
ρ_{calc}g/cm^3	1.423
μ/mm^{-1}	2.865
F(000)	1432.0
Crystal size/mm^3	0.26 × 0.24 × 0.16
Radiation	CuKα (λ = 1.54184)
2Θ range for data collection/°	7.102 to 145.734
Index ranges	-12 ≤ h ≤ 12, -21 ≤ k ≤ 20, -21 ≤ l ≤ 21
Reflections collected	49384
Independent reflections	6413 [R_{int} = 0.0337, R_{sigma} = 0.0143]
Data/restraints/parameters	6413/33/466
Goodness-of-fit on F^2	1.042
Final R indexes [I>=2σ (I)]	R_1 = 0.0559, wR_2 = 0.1573
Final R indexes [all data]	R_1 = 0.0563, wR_2 = 0.1580
Largest diff. peak/hole / e Å^{-3}	1.03/-0.65
Flack parameter	0.007(4)

^{1}H NMR (400 MHz, $CDCl_3$) spectrum of (*aR*)-**A**:

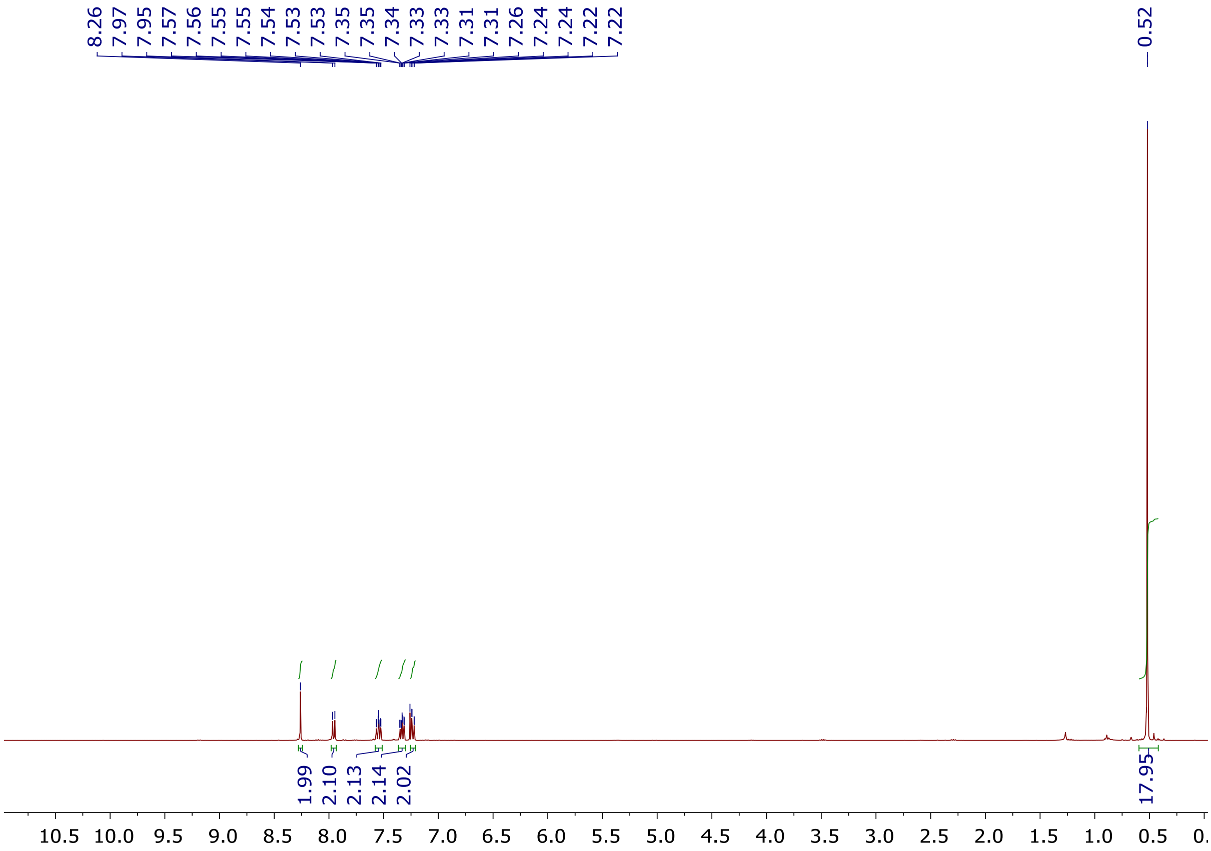

$^{13}C\{^{1}H\}$ NMR (101 MHz, $CDCl_3$) spectra of (*aR*)-**A**:

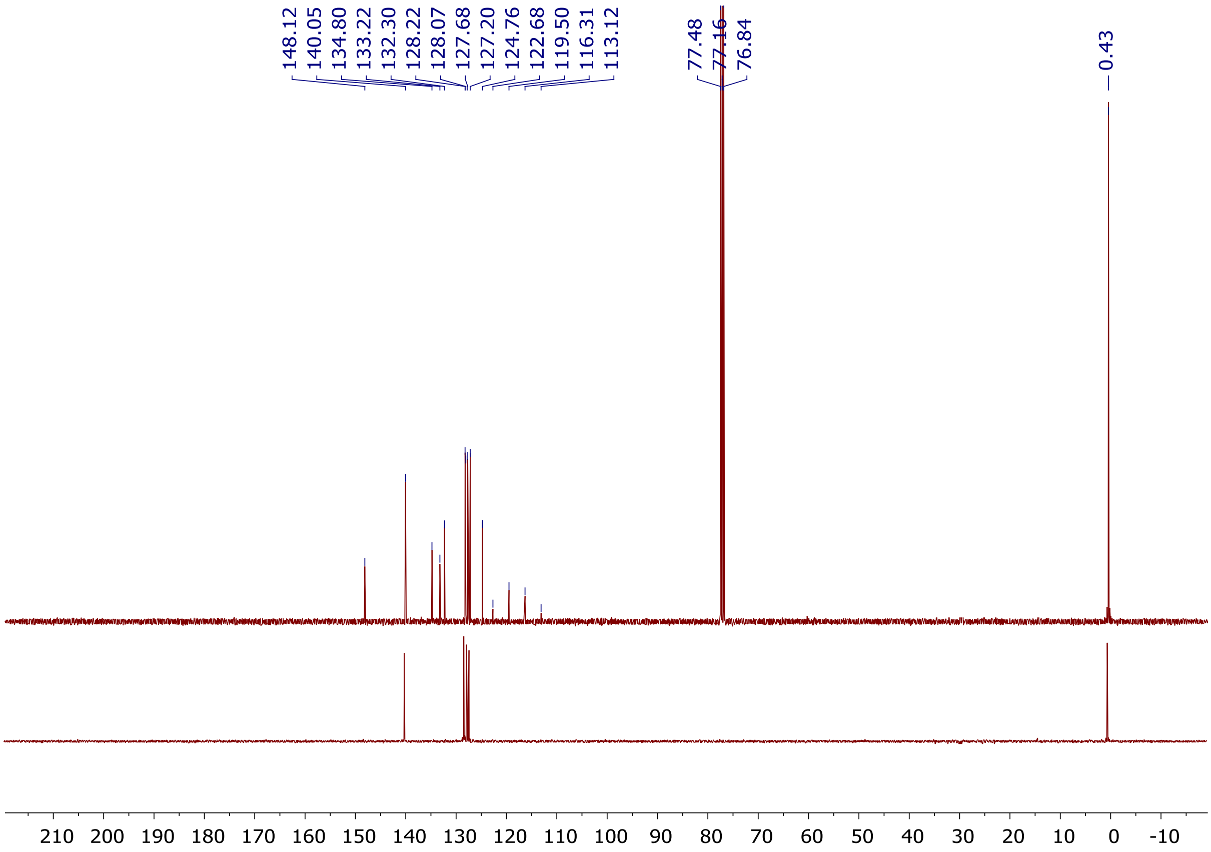

An attempt for the fluoride-induced generation of the bis(aryne) atropisomer synthetic equivalent from (*aR*)-**A** in the presence of 2,3,4,5-tetraphenylcyclopenta-2,4-dien-1-one produced only the known two-fold thia-Fries rearrangement product (*aR*)-**C**[7] in 56% yield after 2 hours with no detectable amount of the expected single- and/or two-fold cycloaddition/decarbonylation product (Scheme S1). A control reaction with benzyne under otherwise identical conditions afforded the expected tetraphenylnaphthalene derivative. Consequently, the Kobayashi-type precursor **A** was abandoned.

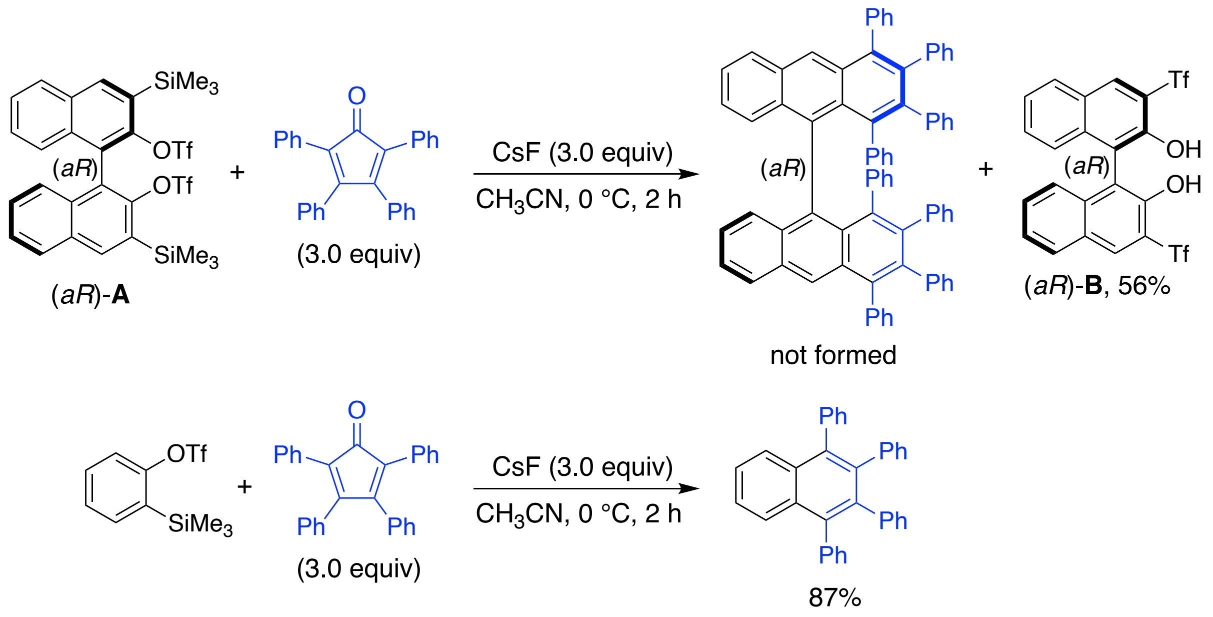

Scheme S1. Attempt for the fluoride-induced generation of the bis(aryne) atropisomer synthetic equivalent from (*aR*)-**A**.

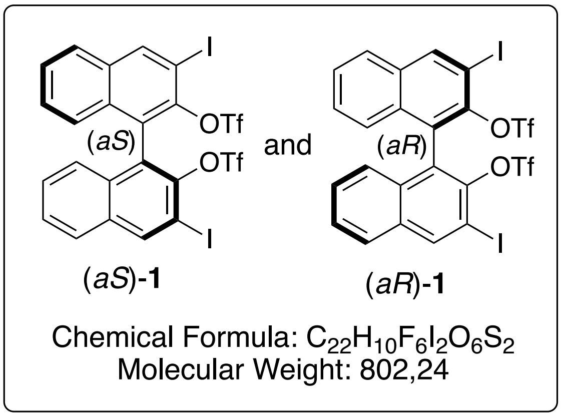

To a solution of (*aS*)-3,3'-diiodo-1,1'-binaphthyl-2,2'-diol (942 mg, 1.75 mmol, >99% *ee*) in anhydrous dichloromethane (20 mL) was added pyridine (0.42 mL, 5.30 mmol) at 0 °C. To the resulting mixture was added Tf_2O (0.88 mL, 5.30 mmol) at this temperature and the reaction was monitored by TLC until the starting material is no longer detectable (2 h) whereupon a

[7] J. P. H. Charmant, A. M. Dyke, G. C. Lloyd-Jones, *Chem. Commun.* **2003**, *2003*, 380–381.

saturated aqueous NH_4Cl solution was added. The resulting mixture was extracted three times with EtOAc. The combined organic layers were dried over anhydrous Na_2SO_4, filtered, and concentrated under reduced pressure to give the crude product. Purification by flash chromatography on silica gel eluted with pentane/EtOAc = 10:1 afforded (*aS*)-**1** (1.39 g, 99%, 99% *ee*) as a light yellow solid. The ratio of enantiomers was obtained by analytical HPLC on a chiral stationary phase. (*aR*)-**1** was obtained in similar yield and *ee* by the same procedure from (*aR*)-3,3'-diiodo-1,1'-binaphthyl-2,2'-diol.

Mp 211–213 °C (amorphous); **TLC** (pentane/EtOAc = 10:1) R*f* = 0.72; **HRMS** (ESI+) *m/z* calcd for $C_{22}H_{14}NO_6S_2F_6I_2$ $[M+NH_4]^+$ 819.8251, found 819.8252; **^{1}H NMR** (400 MHz, $CDCl_3$) δ 8.69 (s, 2H), 7.88 (d, *J* = 8.4 Hz, 2H), 7.59 (ddd, *J* = 8.5, 7.0, 1.0 Hz, 2H), 7.39 (ddd, *J* = 8.9, 7.3, 1.3 Hz, 2H), 7.22 (dd, *J* = 8.6, 0.8 Hz, 2H); **^{13}C{^{1}H} NMR** (101 MHz, $CDCl_3$) δ 145.3 (2C), 143.3 (2CH), 133.8 (2C), 133.4 (2C), 128.4 (2CH), 128.3 (2CH), 127.7 (2CH), 127.3 (2CH), 125.7 (2C), 118.2 (q, $^1J_{C-F}$ = 322.1 Hz, 2CF$_3$), 80.1 (2C); **^{19}F NMR** (376 MHz, $CDCl_3$) δ -72.4; **HPLC** conditions for (*aS*)-**1**: Chiralcel OD3 column, heptane/isopropanol = 80:20, 1 mL/min, 254 nm; retention time: t_{major} = 4.66 min, >99% *ee*; conditions for (*aR*)-**1**: Chiralpak IB column, heptane/ethanol 95:5, 1 mL/min, 254 nm; retention time t_{minor} = 5.65 min, t_{major} = 6.32 min, 99% *ee*; **Specific rotation** for (*aS*)-**1** : $[\alpha]_D^{25}$ = +164.3 (c = 1.0, $CHCl_3$); for (*aR*)-**1** : $[\alpha]_D^{25}$ = -164.3 (c = 1.0, $CHCl_3$).

^{1}H NMR (400 MHz, $CDCl_3$) spectrum of (*aS*)-**1**:

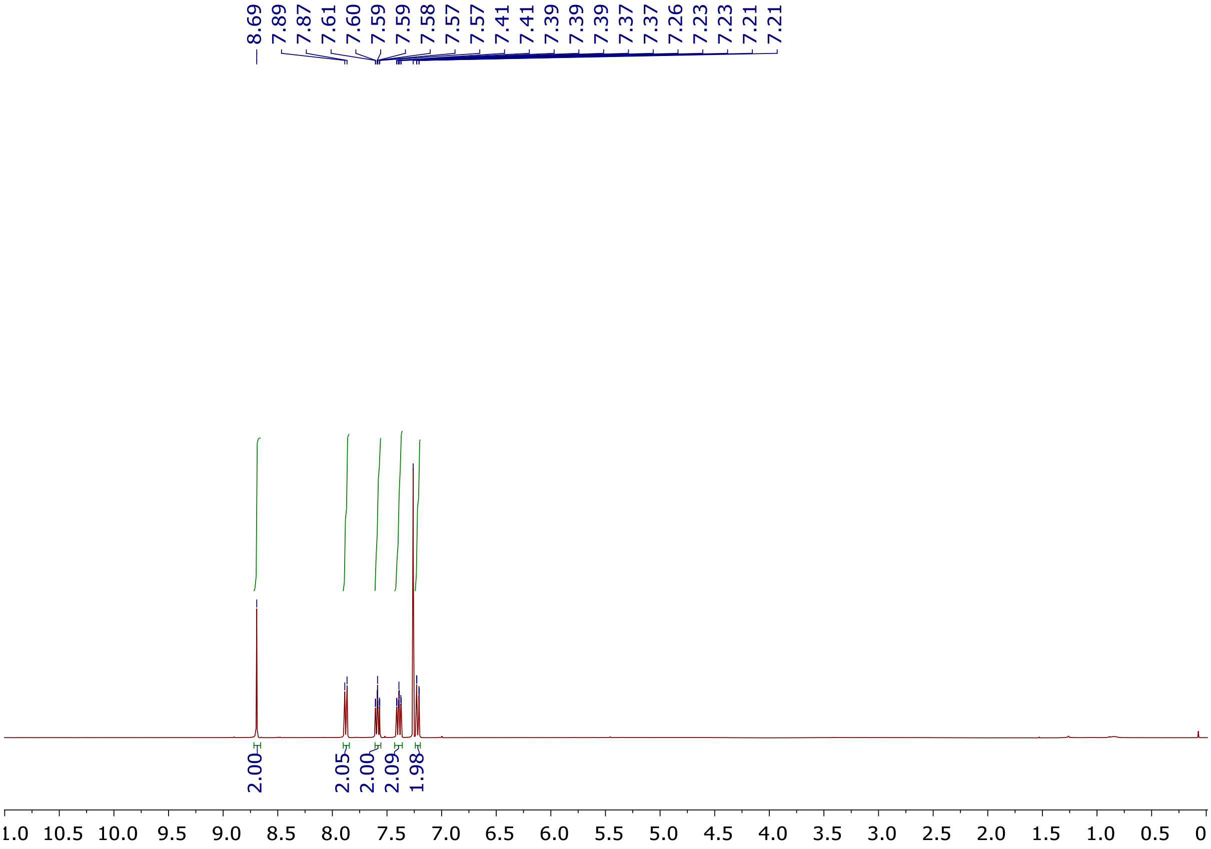

$^{13}C\{^{1}H\}$ NMR (101 MHz, $CDCl_3$) spectra of (*aS*)-**1**:

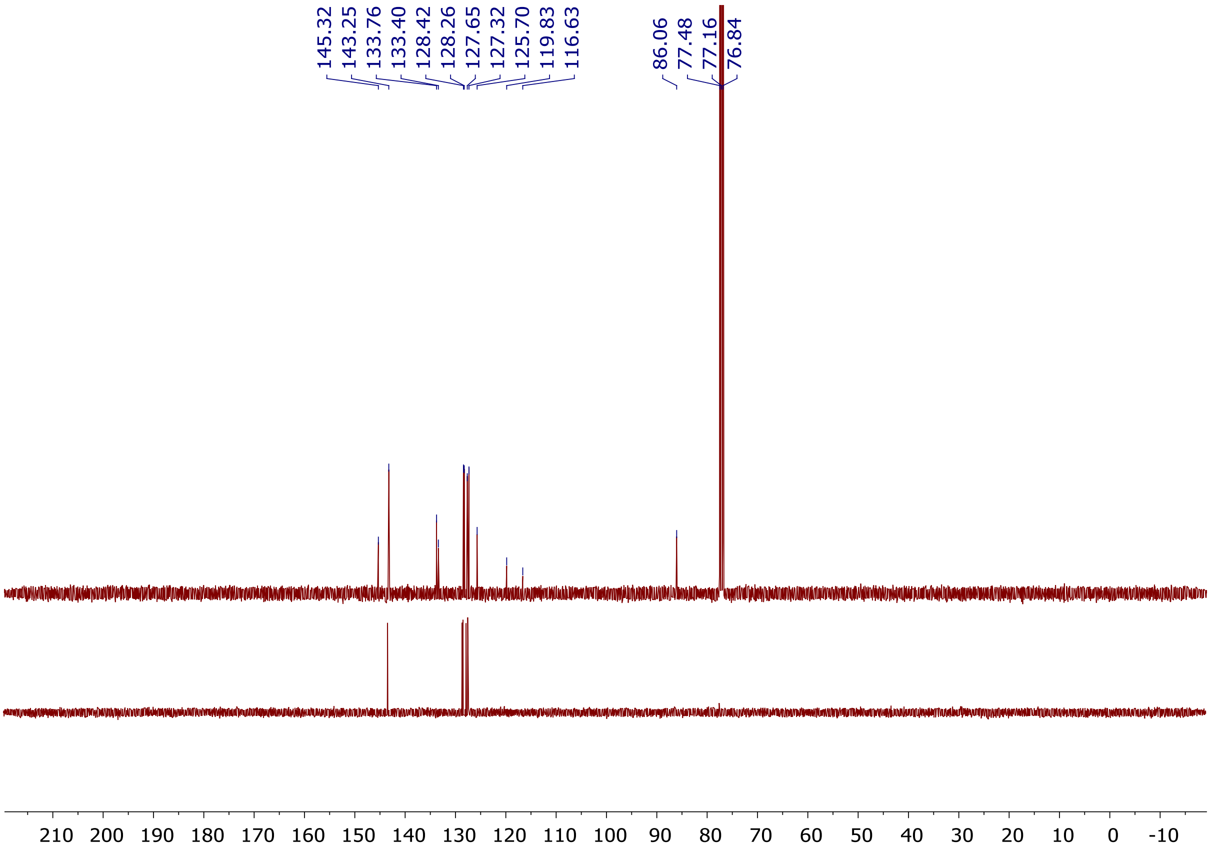

HPLC chromatogram of racemic **1** (Chiralcel OD3 column, heptane/isopropanol = 80:20, 1 mL/min):

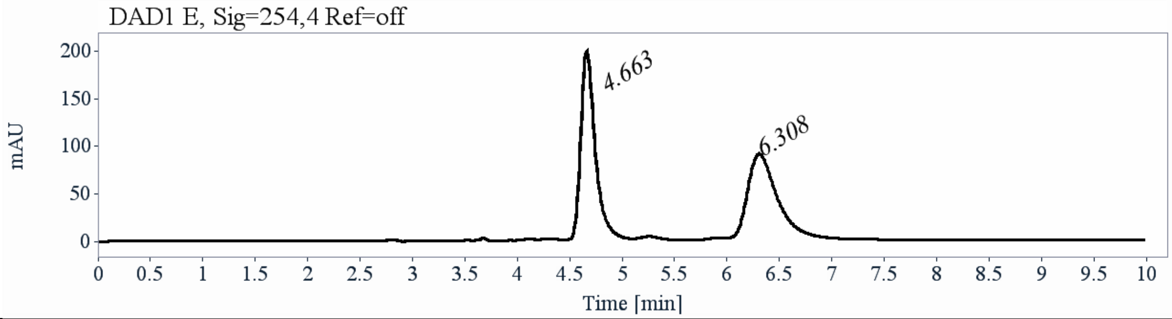

RT [min]	Area	Area%	Capacity Factor	Enantioselectivity	Resolution (USP)
4.66	1907	50.39	0.58		
6.31	1877	49.61	1.14	1.96	4.45
Sum	3784	100.00			

HPLC chromatogram of (*aS*)-**1** (Chiralcel OD3 column, heptane/isopropanol = 80:20, 1 mL/min):

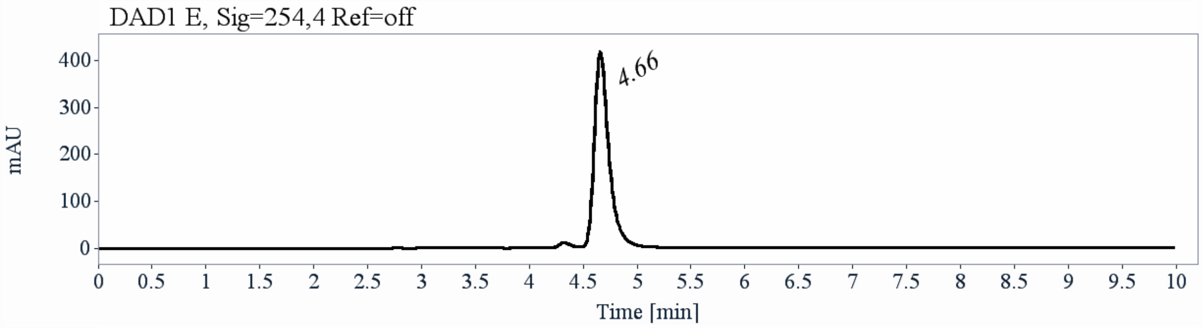

RT [min]	Area	Area%	Capacity Factor	Enantioselectivity	Resolution (USP)
4.66	3958	100.00	0.58		
Sum	3958	100.00			

HPLC chromatogram of racemic **1** (Chiralpak IB column, heptane/ethanol 95:5, 1 mL/min):

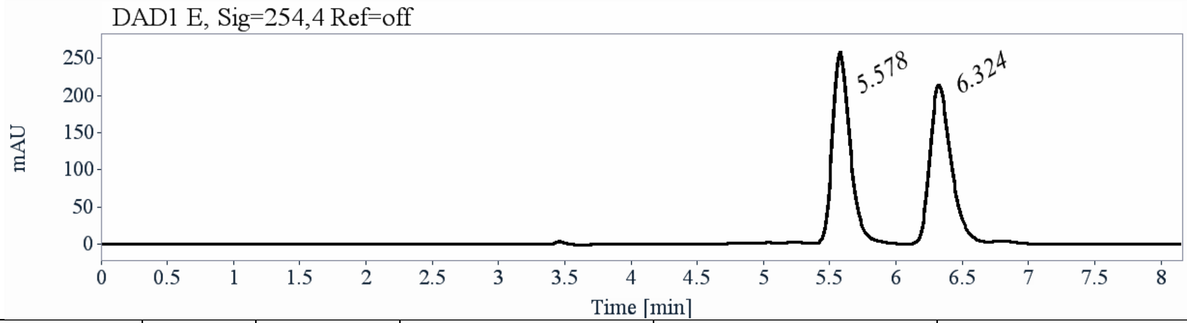

RT [min]	Area	Area%	Capacity Factor	Enantioselectivity	Resolution (USP)
5.58	2386	50.48	0.89		
6.32	2340	49.52	1.14	1.28	2.84
Sum	4726	100.00			

HPLC chromatogram of (*aR*)-**1** (Chiralpak IB column, heptane/ethanol 95:5, 1 mL/min):

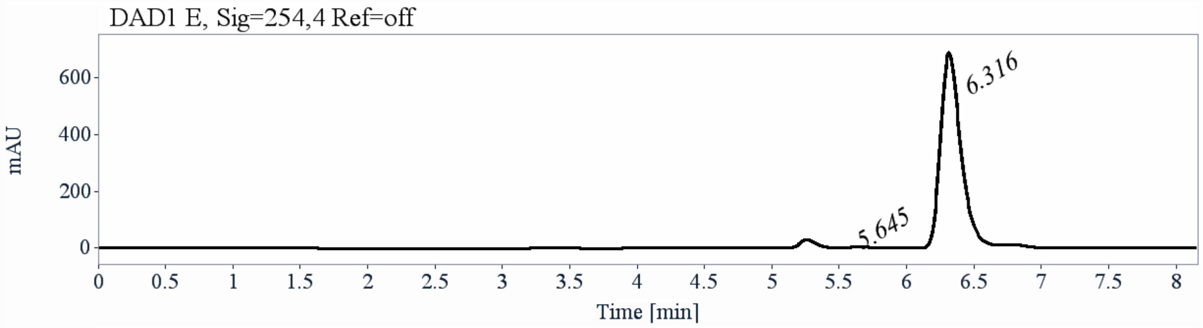

RT [min]	Area	Area%	Capacity Factor	Enantioselectivity	Resolution (USP)
5.65	38	0.53	0.91		
6.32	7065	99.47	1.14	1.25	2.41
Sum	7102	100.00			

General procedure 1: *two-fold cycloaddition of precursor* ***1*** *with arynophiles.*

In a round bottom flask, the aryne precursor (*aS*)-**1** or (*aR*)-**1** (100 mg, 0.12 mmol, 99% *ee*) was solubilized with 3 mL anhydrous toluene, the toluene was evaporated in vacuo, and the

flask was placed under an argon atmosphere (drying step). Then the flask was charged with Et_2O and the arynophile under an argon atmosphere. The suspension was cooled down to 0 °C and trimethylsilylmethylmagnesium chloride (1.0 M in Et_2O, 2.50 mL, 2.50 mmol) was added at this temperature over 4 hours. After stirring for a certain amount of time at 0 °C, the reaction mixture was hydrolyzed with water. The mixture was extracted three times with EtOAc and the combined organic layers were washed with brine, dried over anhydrous Na_2SO_4, filtered and concentrated under reduced pressure to afford the crude product.

General procedure 2: *two-fold deoxygenation of the oxa-bridged intermediates.*
For products (*aS*)-**2** and (*aS*)-**3**, the crude products obtained after the two-fold cycloaddition was directly diluted in anhydrous dichloromethane (2 mL), and NaI (90 mg, 0.60 mmol) and Me_3SiCl (76 µL, 0.60 mmol) were subsequently added to the solution, resulting in an immediate dark brown coloration. The mixture was stirred at 23–25 °C until starting material is no longer detectable by TLC analysis (15 min). The reaction was hydrolyzed with a saturated $NaHCO_3$ aqueous solution and extracted twice with EtOAc. The combined organic layers were dried over anhydrous Na_2SO_4, filtered, and concentrated to afford the crude product.

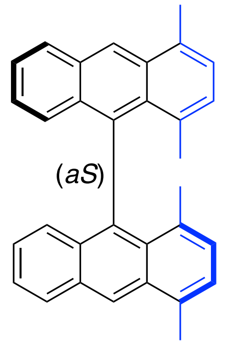

(*aS*)-**2**
Chemical Formula: $C_{32}H_{26}$
Molecular Weight: 410,56

Following the general procedure 1 with (*aS*)-**1** (100 mg, 0.12 mmol, >99% ee) and 2,5-dimethylfuran (53 µL, 0.50 mmol) in 5 mL of Et_2O for 16 hours afforded the crude intermediate oxa-bridged cycloadducts as a white solid (52 mg). This material was directly engaged in the deoxygenation reaction following the general procedure 2. Purification of the resulting crude product by flash chromatography eluted with pentane/Et_2O = 50:1 afforded (*aS*)-**2** (29 mg, 57% after the two steps) as a white solid. **Mp** 245–247 °C (amorphous); **TLC** (pentane/Et_2O = 10:1) R*f* = 0.77; **HRMS** (ESI+) *m/z* calcd for $C^{32}H^{26}Ag^+$ $[M+Ag]^+$ 517.1080, found 517.1081; **^{1}H NMR** (400 MHz, $CDCl_3$) δ 8.79 (s, 2H), 8.09 (d, *J* = 8.3 Hz, 2H), 7.37 (ddd, *J* = 8.5, 6.7, 0.8 Hz, 2H), 7.39 (ddd, *J* = 8.9, 7.3, 1.3 Hz, 2H), 7.2 (d, *J* = 6.9 Hz, 2H), 7.03 (dd, *J* = 6.4, 1.2 Hz, 1H), 7.00 (ddd, *J* = 8.3, 6.4, 1.1 Hz, 3H), 6.80 (dd, *J* = 8.9, 0.9 Hz, 2H), 2.92 (s, 6H), 1.43 (s, 6H); **^{13}C{^{1}H} NMR** (101 MHz, $CDCl_3$) δ 137.3 (2C), 134.4 (2C), 133.4 (2C), 133.0 (2C), 132.2 (2C), 132.1 (2C), 130.3 (2C), 129.1 (2CH), 128.4 (2CH), 127.2 (2CH),125.9 (2CH), 125.8 (2CH), 125.3 (2CH), 124.7 (2CH) 24.8 (2CH_3), 20.8 (2CH_3); **HPLC** Chiralpak IE column, heptane/dichloromethane = 90:10, 1 mL/min, 254 nm, retention time: t_{minor} = 5.35 min, t_{major} = 5.71 min, 99% *ee*; **Specific rotation** $[\alpha]_D^{25}$ = +103.3 (c = 1.0, $CHCl_3$).

The atropisomer (*aR*)-**2** was obtained in similar yield and enantiomeric excess using the same protocol from (*aR*)-**1**. **Specific rotation** $[\alpha]_D^{25}$ = –103.3 (c = 1.0, $CHCl_3$).

^{1}H NMR (400 MHz, CDCl$_3$) spectrum of (*aS*)-**2**:

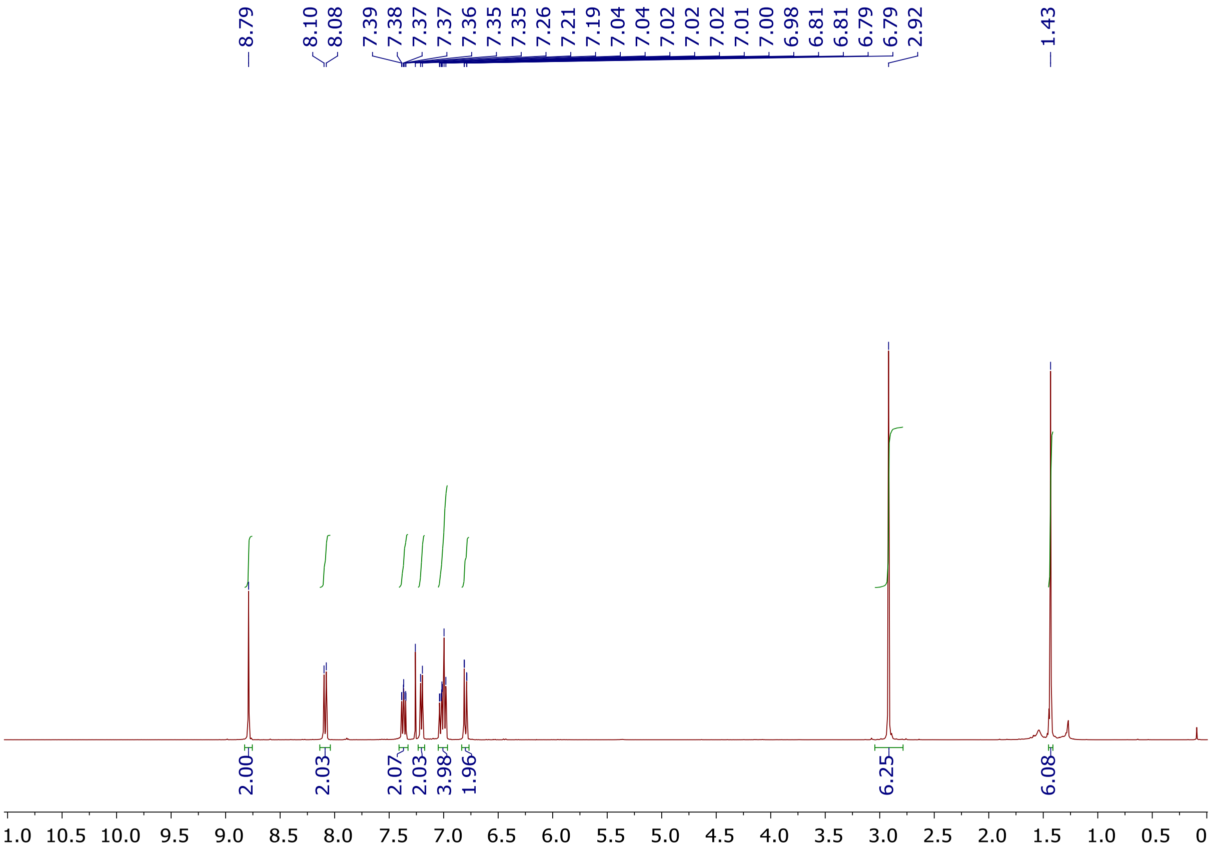

$^{13}C\{^1H\}$ NMR (101 MHz, $CDCl_3$) spectra of (*aS*)-**2**:

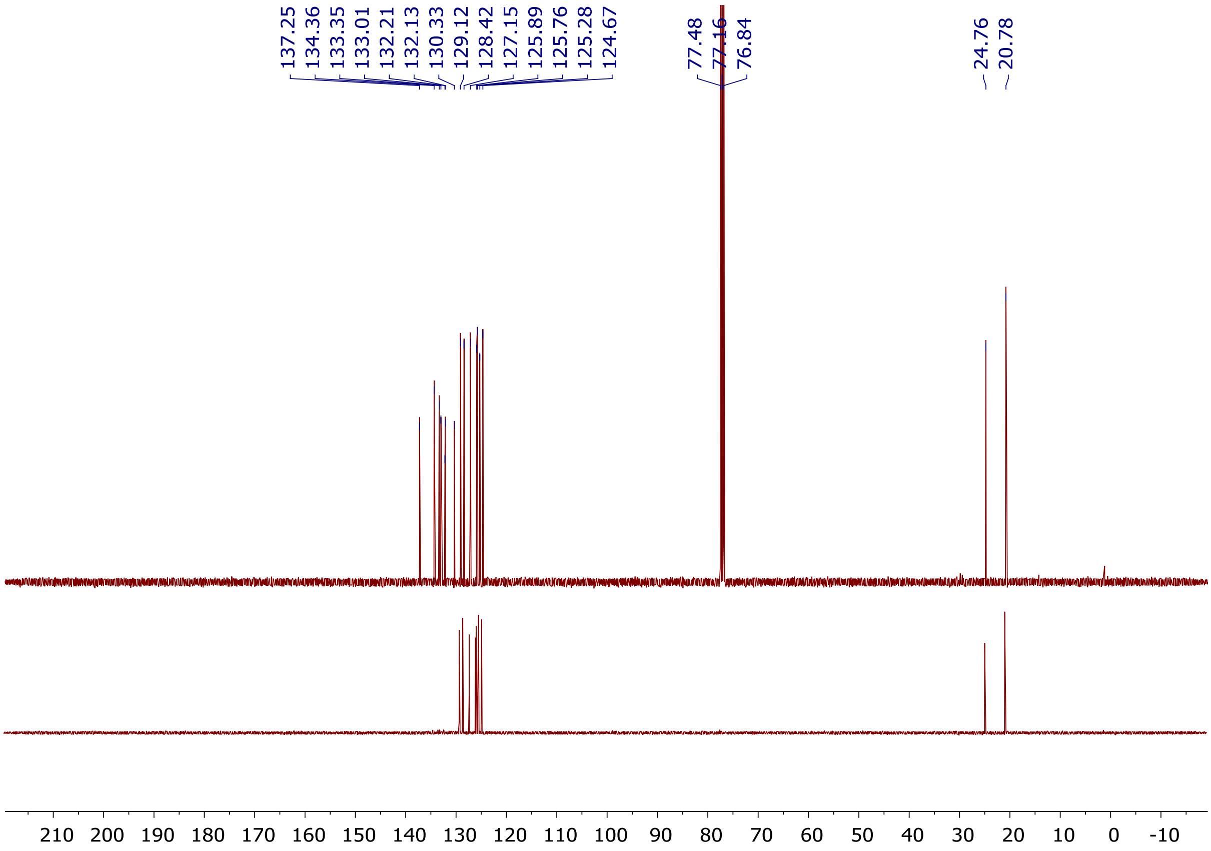

HPLC chromatogram of racemic **2** (Chiralpak IE column, heptane/dichloromethane = 90:10, 1 mL/min):

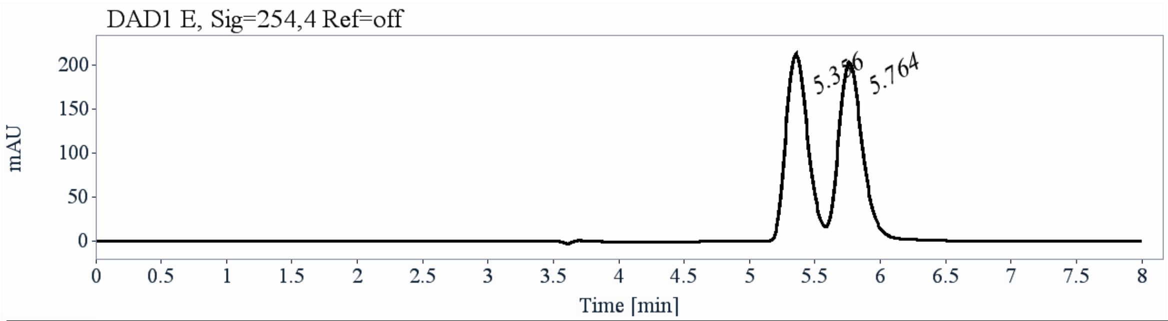

RT [min]	Area	Area%	Capacity Factor	Enantioselectivity	Resolution (USP)
5.36	2472	49.17	0.82		
5.76	2555	50.83	0.95	1.17	1.29
Sum	5027	100.00			

HPLC chromatogram of (*aS*)-**2** (Chiralpak IE column, heptane/dichloromethane = 90:10, 1 mL/min):

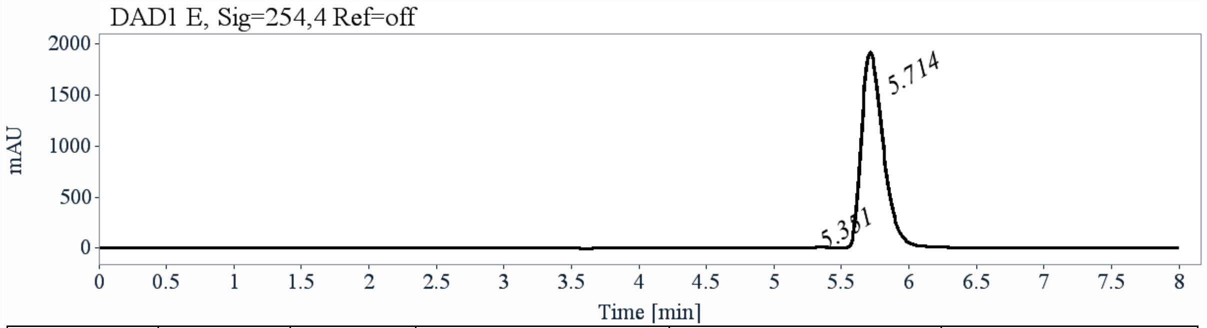

RT [min]	Area	Area%	Capacity Factor	Enantioselectivity	Resolution (USP)
5.35	83	0.38	0.81		
5.71	21520	99.62	0.94	1.15	1.40
Sum	21603	100.00			

Solutions of (*aS*)-**2** and (*aR*)-**2** with a concentration of 0.15 mmol·L^{-1} were prepared in acetonitrile (HPLC grade). The CD spectrometer was purged with nitrogen during the recording of spectra. The UV absorption and ECD spectra were recorded using acetonitrile as a reference and are presented without smoothing and further data processing (Figure S2). Atropisomer (*aS*)-**2** exists in a single conformation which was optimized with Gaussian16 using DFT at the SMD(acetonitrile)/B3LYP-GD3BJ/6-311G(d,p) level of theory.[9,10,13,18b]
Based on the optimized geometry, the ECD and UV spectra were calculated using time dependent density functional theory with SMD(acetonitrile)/CAM-B3LYP/6-31++G(d,p). Calculations were performed for vertical 1A singlet excitation using 60 states. For a comparison between theoretical results and the experimental values, the calculated UV and ECD spectra have been modeled with a gaussian function, using a half-width of 0.37 eV. Due to the approximations of the theoretical model used, an offset almost constant was observed between measured and calculated frequencies. Using UV spectra, all frequencies were calibrated by a factor of 1.02.
An excellent agreement was found between the experimental and simulated spectra of (*aS*)-**2**, confirming its absolute configuration.

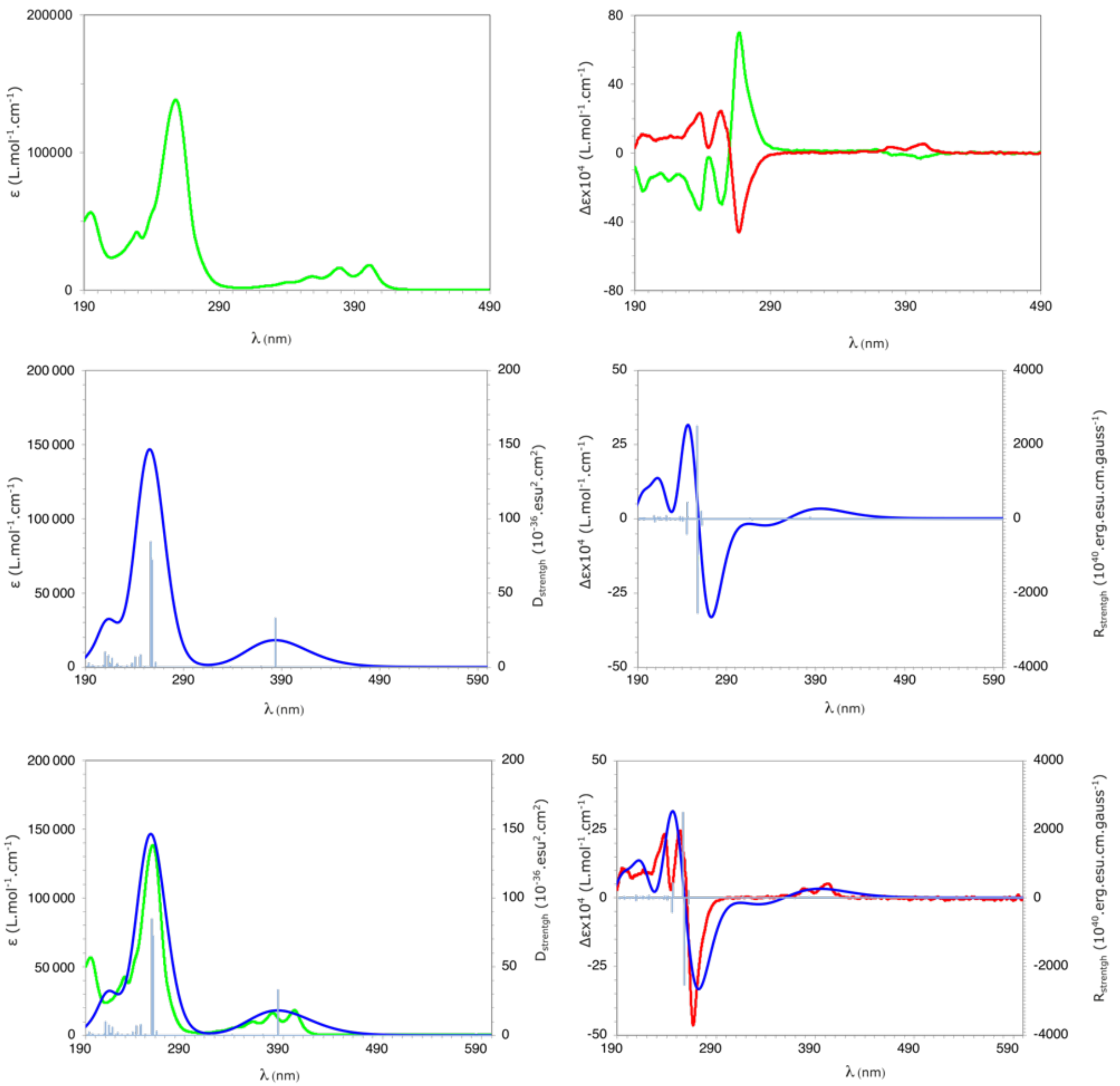

Figure S2. UV (left) and ECD (right) spectroscopic analyses of (*aS*)-**2** and (*aR*)-**2**. In green color: experimental spectra of (*aR*)-**2** recorded in CH_3CN; in red color: experimental spectra of (*aS*)-**2** recorded in CH_3CN; in blue color: simulated spectra for (*aS*)-**2** using SMD(acetonitrile)/CAM-B3LYP/6-31++G(d,p)//SMD(acetonitrile)/B3LYP-D3BJ/6-311Gd,p).

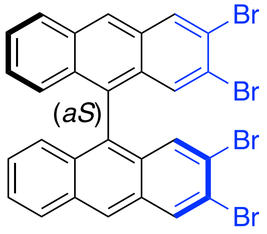

(aS)-**3**

Chemical Formula: $C_{28}H_{14}Br_4$
Molecular Weight: 670,04

3,4-Dibromofuran was prepared by a known procedure.[8] Following the general procedure 1 with (*aS*)-**1** (100 mg, 0.12 mmol, >99% *ee*) and 3,4-dibromofuran (52 µL, 0.50 mmol) in 5 mL of Et_2O for 16 hours afforded the crude intermediate oxa-bridged cycloadducts as a white solid (63 mg). This material was directly engaged in the deoxygenation reaction following the general procedure 2. Purification of the resulting crude product by flash chromatography eluted with pentane/Et_2O = 50:1 afforded (*aS*)-**3** (43 mg, 52% after two steps) as a yellow solid. Recrystallization of (*aS*)-**3** from chloroform/methanol (1:1, slow evaporation) afforded monocrystalline needles suitable for X-ray diffraction analysis, which confirmed both the structure and the absolute configuration of (*aS*)-**3** (Figure S3, Table S2, CCDC 2173781). **Mp** 324–326 °C ($CHCl_3$/MeOH 1:1); **TLC** (pentane/Et_2O = 10:1) R*f* = 0.59; **HRMS** (ESI+) *m/z* calcd for $C_{28}H_{15}Br_4^+$ $[M+H]^+$ 670.7864, found 670.7865; **^{1}H NMR** (400 MHz, $CDCl_3$) δ 8.59 (s, 2H), 8.49 (s, 2H), 8.14 (d, *J* = 8.5 Hz, 2H), 7.50 (ddd, *J* = 9.1, 6.8, 1.1 Hz, 2H), 7.32 (d, *J* = 0.4 Hz, 2H), 7.22 (dd, *J* = 6.5, 1.2 Hz, 2H), 7.19 (dd, *J* = 6.5, 1.2 Hz, 1H), 6.94 (dd, *J* = 8.9, 0.9 Hz, 2H); **^{13}C{^{1}H} NMR** (101 MHz, $CDCl_3$) δ 133.0 (2CH), 132.3 (2C), 132.3 (2C), 131.4 (2C), 131.0 (2C), 130.9 (2C), 130.6 (2CH), 128.9 (2CH), 127.4 (2CH), 127.2 (2CH),126.6 (2CH), 126.6 (2CH), 123.1 (2C), 122.2 (2C); **HPLC** Lux-Amylose-1 column, heptane/isopropanol = 90:10, 1 mL/min, 254 nm, retention time: t_{major} = 5.05 min, t_{minor} = 7.21 min, 98% *ee*; **Specific rotation** $[\alpha]_D^{25}$ = +136.9 (c = 1.0, $CHCl_3$).

[8] G. A. Kraus, X. Wang, *Synth. Commun.* **1998**, *28*, 1093–1096.

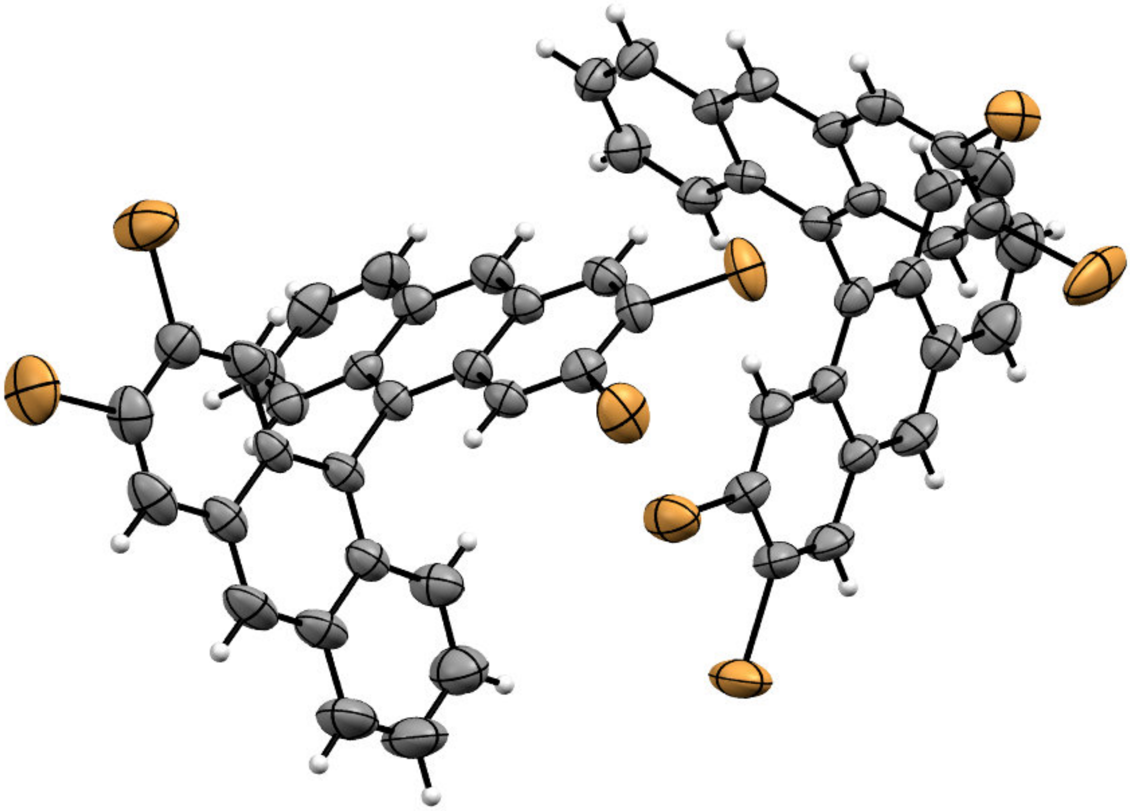

Figure S3. ORTEP representation of (*aS*)-**3** obtained by single-crystal X-ray diffraction analysis. The ellipsoids are drawn at the 50% probability level and H atoms are represented as fixed-size spheres of 0.15 Å radius. These measurements allowed the confirmation of the absolute configuration in (*aS*)-**3**.

Table S2. Crystal data and structure refinement for (*aS*)-**3**.

Empirical formula	$C_{56}H_{28}Br_8$
Formula weight	665.78
Temperature/K	295
Crystal system	orthorhombic
Space group	$P2_12_12_1$
a/Å	12.64895(14)
b/Å	21.1101(2)
c/Å	21.9276(2)
Volume/Å^3	5855.12(11)
Z	4
ρ_{calc}g/cm^3	1.907
μ/mm^{-1}	10.369
F(000)	3256.0
Crystal size/mm^3	0.18 × 0.14 × 0.12
Radiation	Cu Kα (λ = 1.54184)

2Θ range for data collection/°	5.812 to 145.636
Index ranges	-15 ≤ h ≤ 15, -23 ≤ k ≤ 26, -27 ≤ l ≤ 26
Reflections collected	43668
Independent reflections	11453 [R_{int} = 0.0388, R_{sigma} = 0.0261]
Data/restraints/parameters	11453/24/688
Goodness-of-fit on F^2	1.042
Final R indexes [I>=2σ (I)]	R_1 = 0.0455, wR_2 = 0.1264
Final R indexes [all data]	R_1 = 0.0473, wR_2 = 0.1289
Largest diff. peak/hole / e Å^{-3}	0.92/-0.60
Flack parameter	-0.014(7)

^{1}H NMR (400 MHz, $CDCl_3$) spectrum of (*aS*)-**3**:

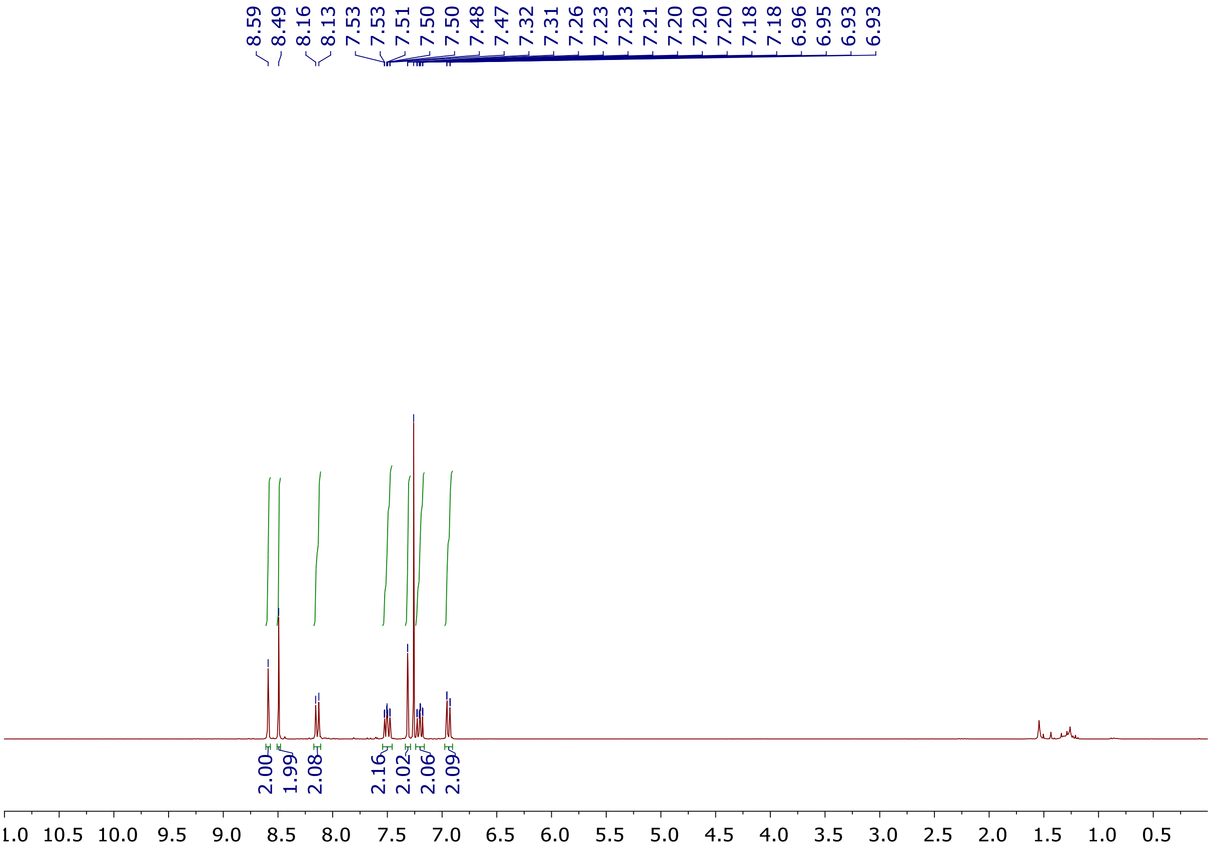

$^{13}C\{^{1}H\}$ NMR (101 MHz, $CDCl_3$) spectra of (*aS*)-**3**:

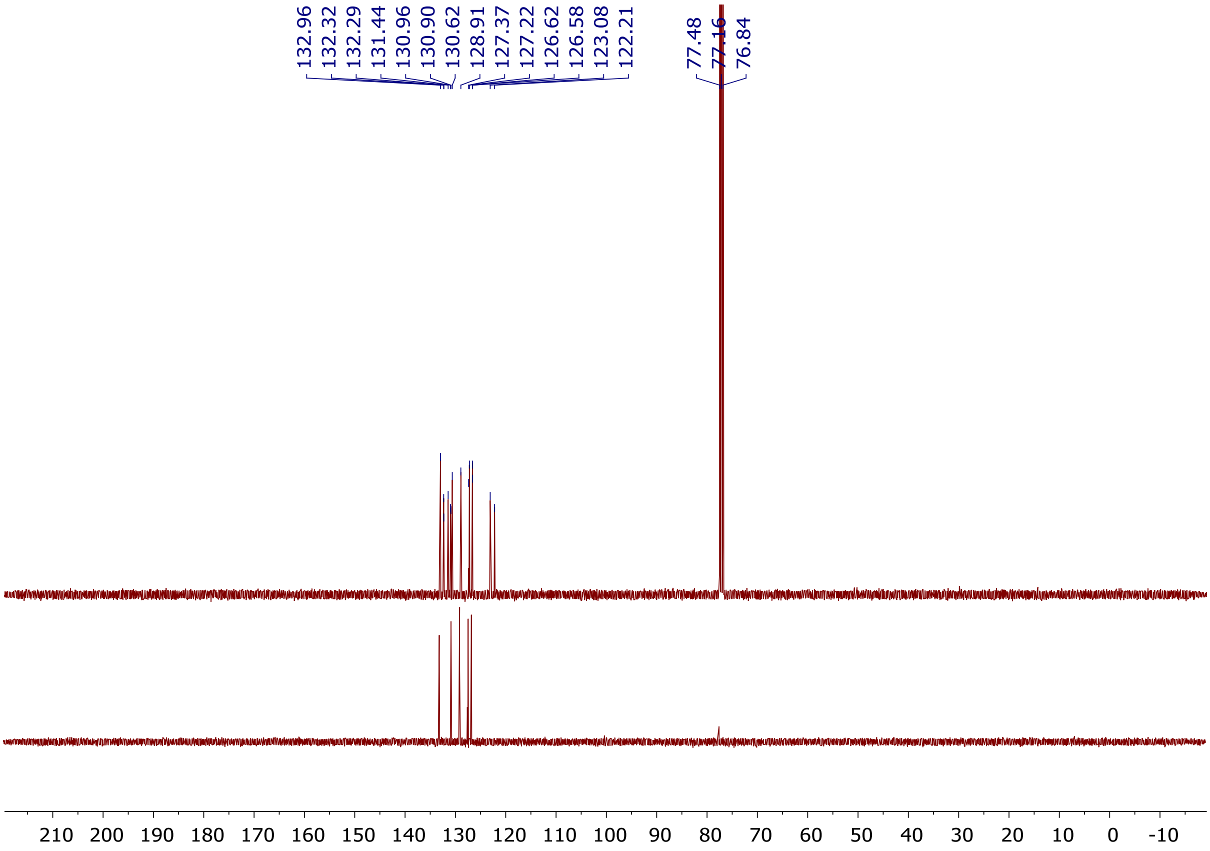

HPLC chromatogram of racemic **3** (Lux-Amylose-1 column, heptane/isopropanol = 90:10, 1 mL/min):

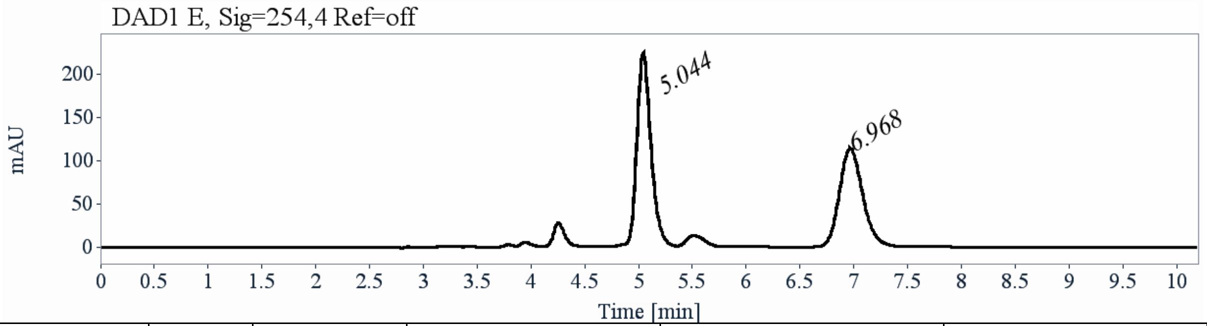

RT [min]	Area	Area%	Capacity Factor	Enantioselectivity	Resolution (USP)
5.04	1892	50.58	0.71		
6.97	1849	49.42	1.36	1.92	6.13
Sum	3741	100.00			

HPLC chromatogram of (*aS*)-**3** (Lux-Amylose-1 column, heptane/isopropanol = 90:10, 1 mL/min):

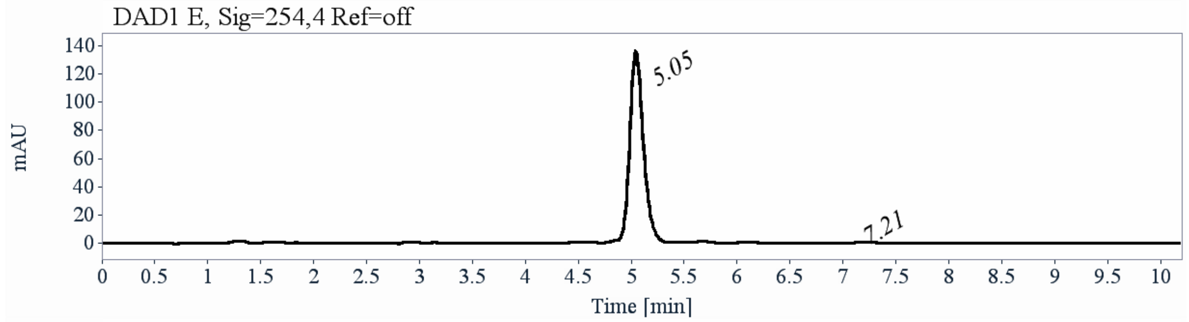

RT [min]	Area	Area%	Capacity Factor	Enantioselectivity	Resolution (USP)
5.05	1228	98.90	0.71		
7.21	14	1.10	1.44	2.03	7.55
Sum	1242	100.00			

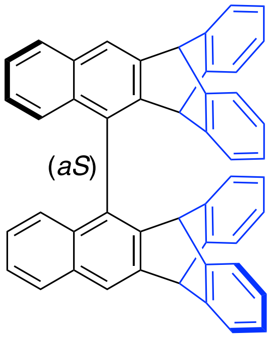

(*aS*)-**4**
Chemical Formula: $C_{48}H_{30}$
Molecular Weight: 606,77

Following the general procedure 1 with (*aS*)-**1** (100 mg, 0.12 mmol, >99% *ee*) and anthracene (110 mg, 0.50 mmol) in 5 mL of Et_2O after 16 hours afforded the crude product. Purification by flash column chromatography eluted with pentane/Et_2O = 10:1 afforded the bis(triptycene) atropisomer (*aS*)-**4** (43 mg, 57%) as a white solid. Recrystallization of (*aS*)-**4** from chloroform/methanol (1:1, slow evaporation) afforded monocrystalline needles suitable for X-ray diffraction analysis, which confirmed both the structure and the absolute configuration of (*aS*)-**4** (Figure S4, Table S3, CCDC 2173779). **Mp** 223–225 °C ($CHCl_3$/MeOH 1:1); **TLC** (pentane/Et_2O = 10:1) R*f* = 0.64; **HRMS** (ESI+) *m/z* calcd for $C_{48}H_{30}Ag^+$ $[M+Ag]^+$ 715.1399, found 715.1397; ^{1}**H NMR** (400 MHz, $CDCl_3$) δ 8.04 (s, 2H), 7.89 (d, *J* = 8.0 Hz, 2H), 7.50 (d, *J* = 7.5 Hz, 2H), 7.47 (d, *J* = 7.4 Hz, 2H), 7.42 (ddd, *J* = 9.3, 7.0, 1.0 Hz, 2H), 7.10–7.00 (m, 6H), 6.92 (ddd, *J* = 8.5, 6.6, 0.8 Hz, 2H), 6.87 (ddd, *J* = 8.5, 6.6, 0.8 Hz, 2H), 6.78 (d, *J* = 8.7 Hz, 2H), 6.59 (d, *J* = 7.4 Hz, 2H), 6.52 (d, *J* = 7.4 Hz, 2H), 5.70 (s, 2H), 4.71 (s, 2H); ^{13}**C{^{1}H} NMR** (101 MHz, $CDCl_3$) δ 145.0 (2C), 145.0 (2C), 144.1 (2C), 143.9 (2C), 142.4 (2C), 141.9 (2C), 132.0

(2C), 131.3 (2C), 129.4 (2C), 127.8 (2CH), 127.0 (2CH), 126.0 (4CH), 125.7 (2CH), 125.5 (2CH) 125.3 (2CH), 125.3 (2CH), 125.1 (2CH), 125.0 (2CH), 123.5 (2CH), 123.5 (2CH), 122.1 (2CH), 54.5 (2CH), 51.3 (2CH); **HPLC** Chiralpak IB N-5 column, heptane/dichloromethane = 80:20, 1 mL/min, 310 nm, retention time: t_{major} = 10.32 min, t_{minor} = 12.50 min, 99% *ee*; **Specific rotation** $[\alpha]_D^{25}$ = +76.9 (c = 1.0, $CHCl_3$).

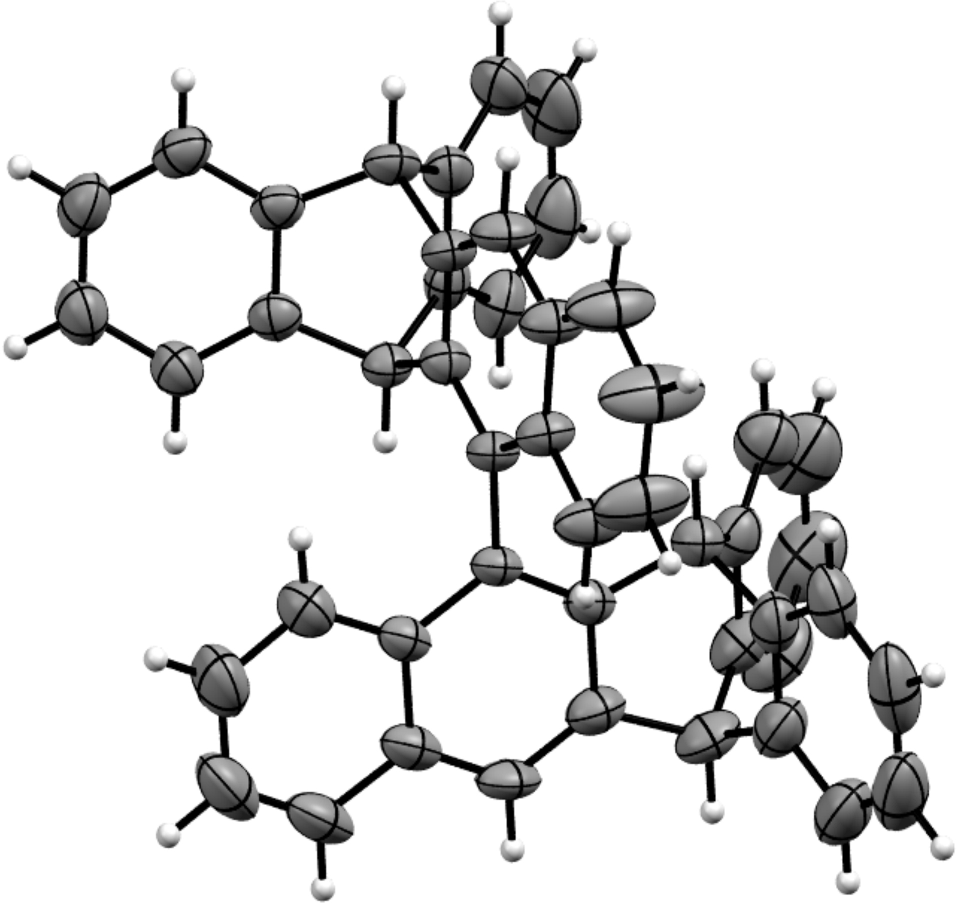

Figure S4. ORTEP representation of (*aS*)-**4** obtained by single-crystal X-ray diffraction analysis. The ellipsoids are drawn at the 50% probability level and H atoms are represented as fixed-size spheres of 0.15 Å radius. These measurements allowed the confirmation of the absolute configuration in (*aS*)-**4**.

Table S3. Crystal data and structure refinement for (*aS*)-**4**.

Empirical formula	$C_{48}H_{30}$
Formula weight	606.23
Temperature/K	295
Crystal system	orthorhombic
Space group	$P2_12_12_1$
a/Å	9.35513(15)
b/Å	17.50883(17)
c/Å	22.9684(3)

Volume/Å^3	3762.16(8)
Z	4
ρ_{calc}g/cm^3	1.281
μ/mm^{-1}	2.451
F(000)	1503.0
Crystal size/mm^3	0.2 × 0.16 × 0.03
Radiation	Cu Kα (λ = 1.54184)
2Θ range for data collection/°	6.348 to 145.676
Index ranges	-11 ≤ h ≤ 11, -21 ≤ k ≤ 8, -28 ≤ l ≤ 28
Reflections collected	19810
Independent reflections	7259 [R_{int} = 0.0371, R_{sigma} = 0.0384]
Data/restraints/parameters	7259/26/497
Goodness-of-fit on F^2	1.047
Final R indexes [I>=2σ (I)]	R_1 = 0.0575, wR_2 = 0.1590
Final R indexes [all data]	R_1 = 0.0713, wR_2 = 0.1706
Largest diff. peak/hole / e Å^{-3}	0.38/-0.43
Flack parameter	-0.011(8)

^{1}H NMR (400 MHz, $CDCl_3$) spectrum of (*aS*)-**4**:

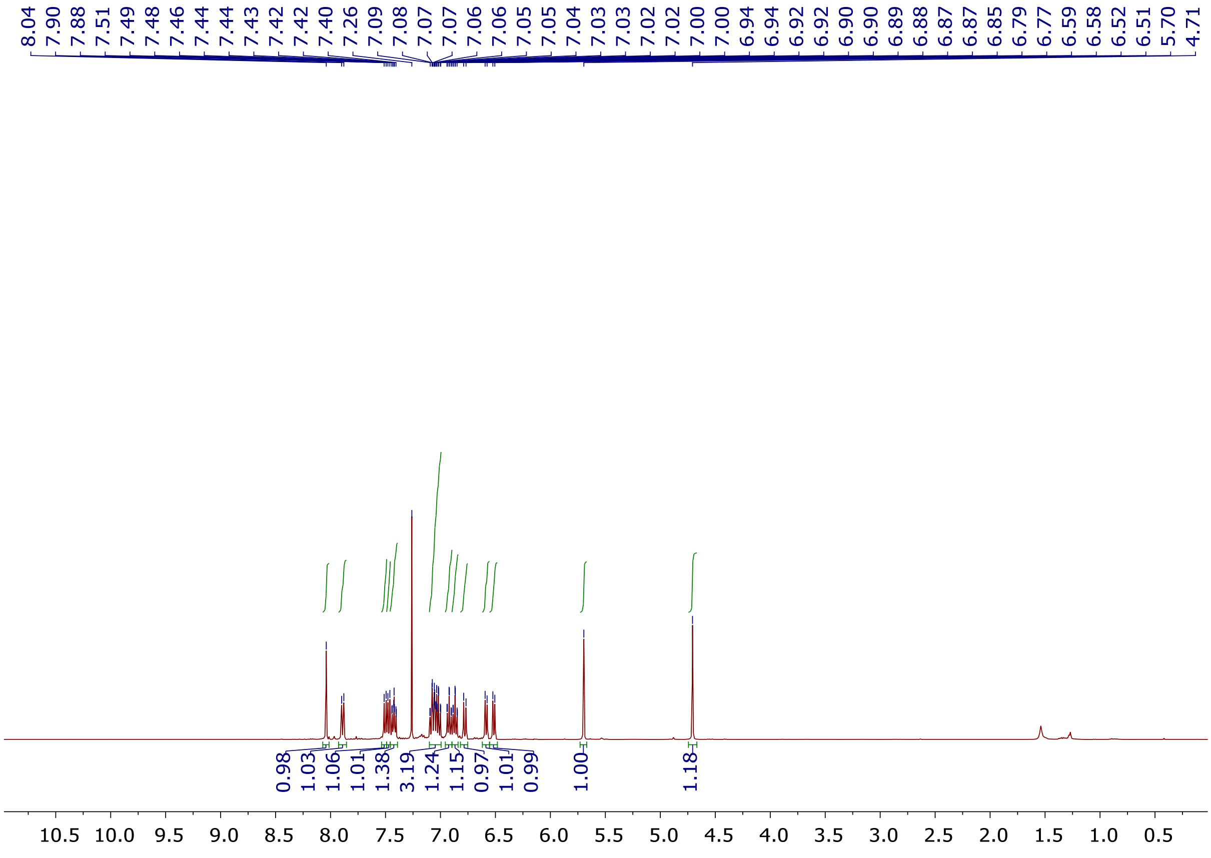

$^{13}C\{^1H\}$ NMR (101 MHz, $CDCl_3$) spectra of (*aS*)-**4**:

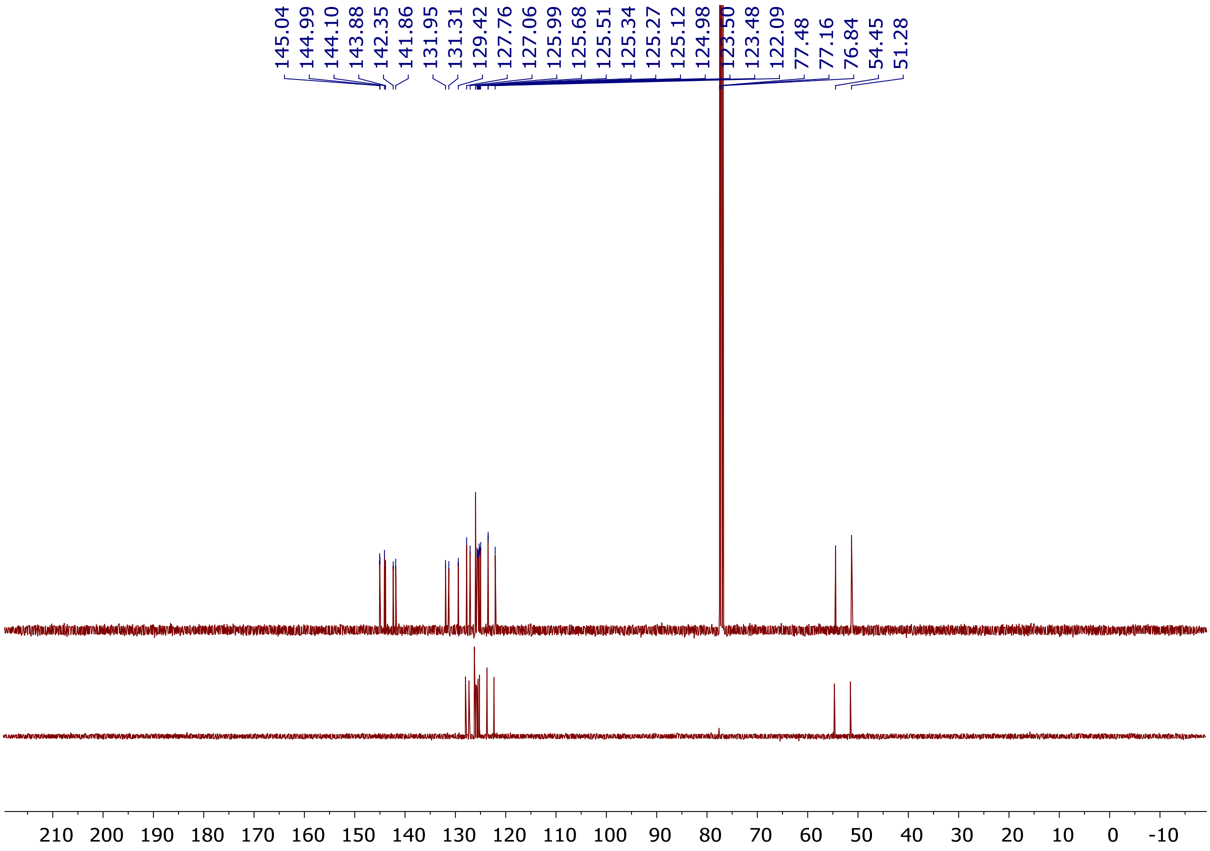

HPLC chromatogram of racemic **4** (Chiralpak IB N-5 column, heptane/dichloromethane = 80:20, 1 mL/min):

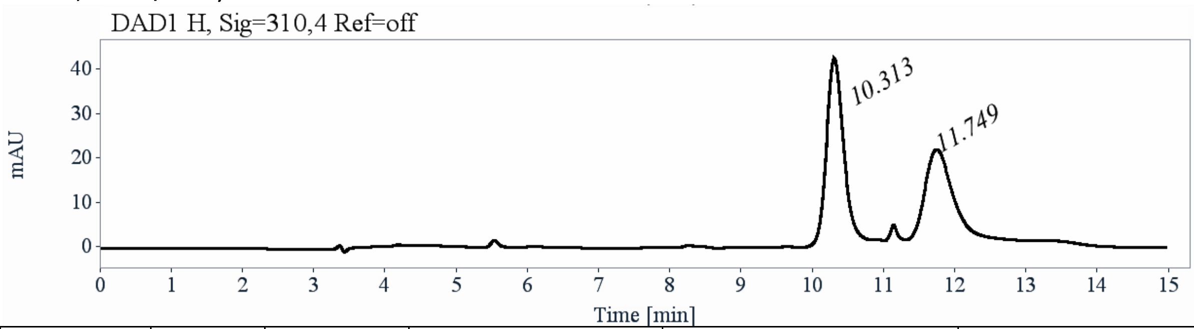

RT [min]	Area	Area%	Capacity Factor	Enantioselectivity	Resolution (USP)
10.31	683	50.44	2.50		
11.75	671	49.56	2.98	1.20	2.50
Sum	1354	100.00			

HPLC chromatogram of (*aS*)-**4** (Chiralpak IB N-5 column, heptane/dichloromethane = 80:20, 1 mL/min):

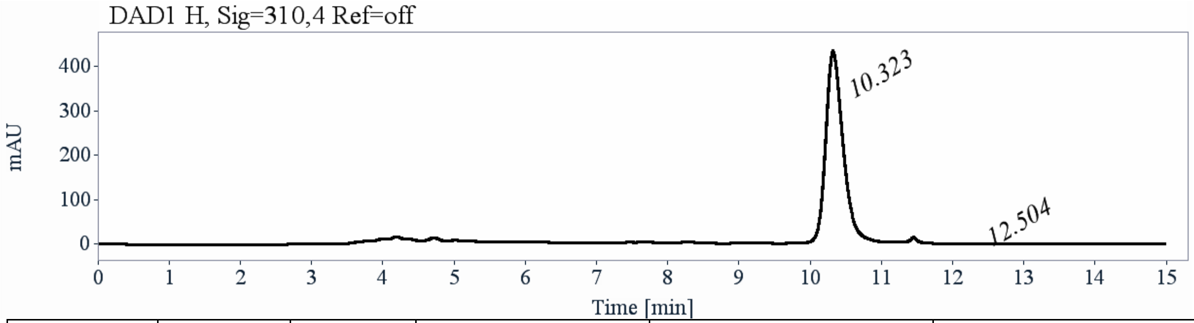

RT [min]	Area	Area%	Capacity Factor	Enantioselectivity	Resolution (USP)
10.32	7490	99.46	2.50		
12.50	441	0.54	3.24	1.30	2.45
Sum	7531	100.00			

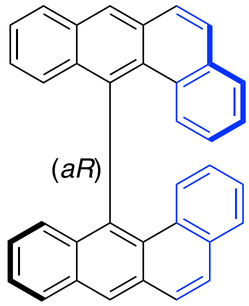

(*aR*)-**5**

Chemical Formula: $C_{36}H_{22}$
Molecular Weight: 454,57

Following the general procedure 1 with (*aR*)-**1** (100 mg, 0.12 mmol, 99% *ee*) and 2-bromostyrene (32 µL, 0.50 mmol) in 5 mL of Et_2O after 16 hours afforded the crude product. Purification by flash column chromatography eluted with pentane/Et_2O = 10:1 afforded (*aR*)-**5** (20 mg, 37%) as a white solid. **Mp** 269–271 °C (amorphous); **TLC** (pentane/Et_2O = 10:1) R*f* = 0.65; **HRMS** (ESI+) *m/z* calcd for $C_{60}H_{30}Ag^+$ $[M+Ag]^+$ 563.0769, found 563.0768; **^{1}H NMR** (400 MHz, $CDCl_3$) δ 8.68 (s, 2H), 8.12 (d, *J* = 8.8 Hz, 2H), 8.00 (d, *J* = 8.5 Hz, 2H), 7.68 (dd, *J* = 7.3, 1.5 Hz, 2H), 7.68 (d, *J* = 9.5 Hz, 2H), 7.44 (d, *J* = 9.5 Hz, 2H), 7.42 (ddd, *J* = 7.8, 6.1, 1.1 Hz, 2H), 7.23 (ddd, *J* = 8.3, 7.7, 1.0 Hz, 2H), 7.00 (dd, *J* = 6.8,1.3 Hz 1H), 6.98 (dd, *J* = 6.9, 1.3 Hz, 1H), 6.87 (dd, *J* = 8.7, 0.8 Hz, 2H), 6.65 (ddd, *J* = 8.8, 7.1, 1.5 Hz, 2H); the regioselectivity was established by 2D ^{1}H NMR experiments (COSY and NOESY); **^{13}C{^{1}H} NMR** (101 MHz, $CDCl_3$) δ 137.1 (2C), 133.6 (2C), 132.3 (2C), 132.1 (2C), 132.0 (2C), 131.2 (2C), 128.7 (2C), 128.6 (4CH), 128.5 (2CH), 128.2 (2CH), 127.8 (2CH), 127.2 (2CH), 126.6 (2CH), 126.5 (4CH), 126.4 (2CH), 126.2 (2CH); **HPLC** Chiralpak IB N-5 column, heptane/dichloromethane = 95:5, 1 mL/min, 254 nm, retention time: t_{major} = 16.21 min, t_{minor} = 14.95 min, 99% *ee*; **Specific rotation** $[\alpha]_D^{25}$ = -250.7 (c = 1.0, $CHCl_3$).

^{1}H NMR (400 MHz, $CDCl_3$) spectra of (*aR*)-**5**, including COSY and NOESY:

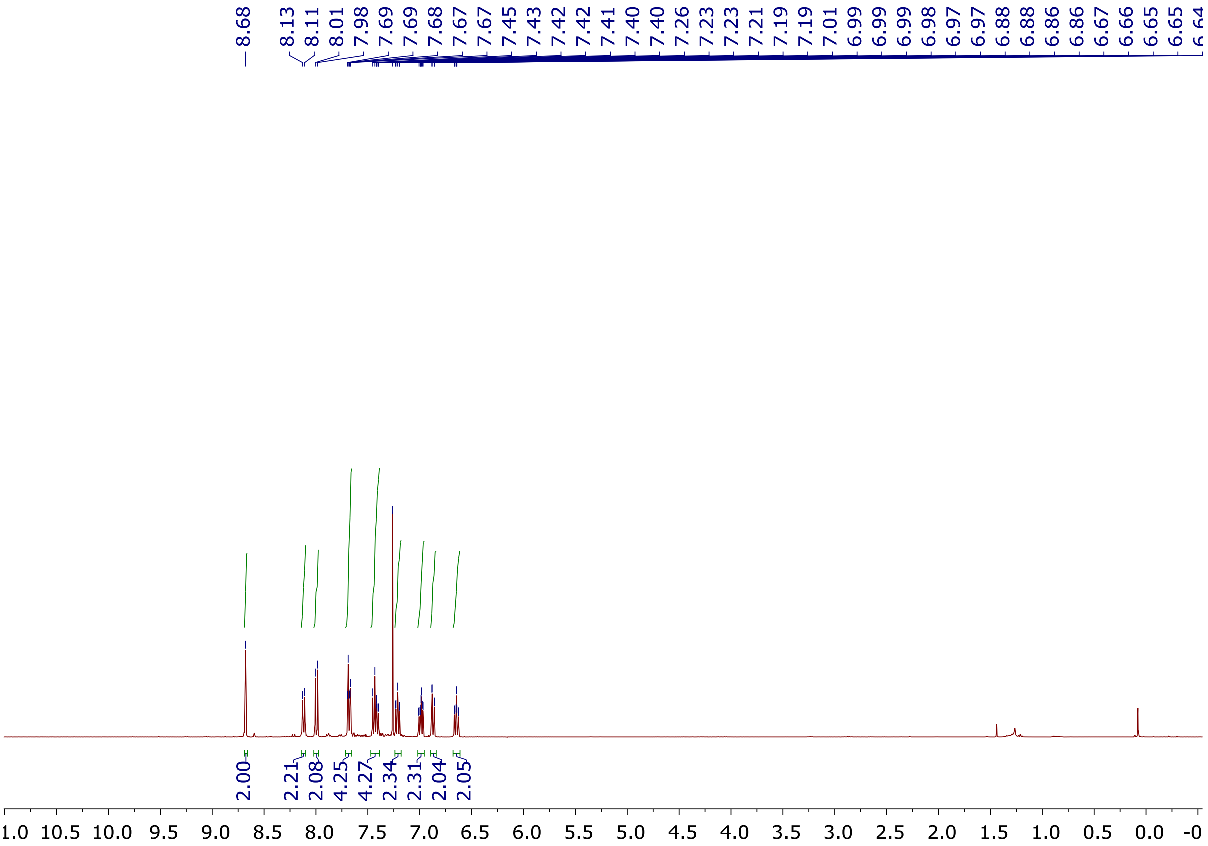

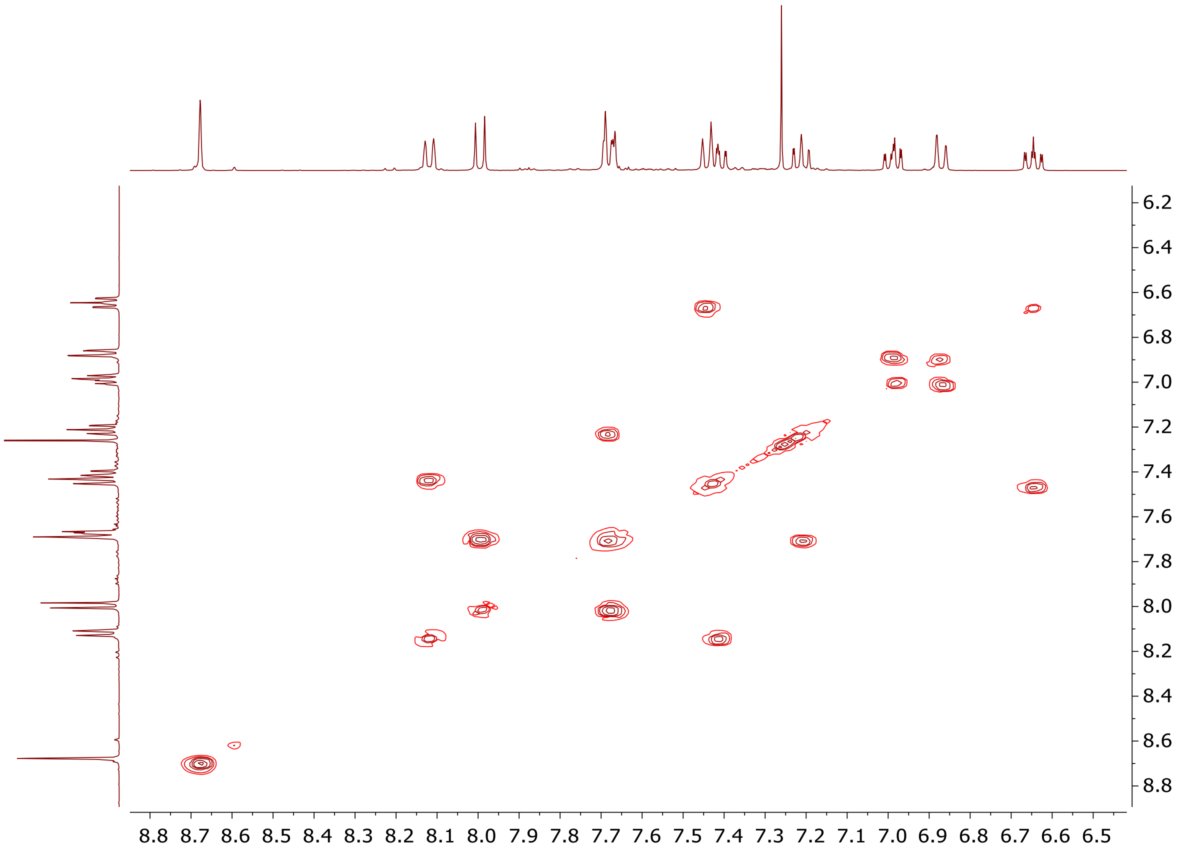

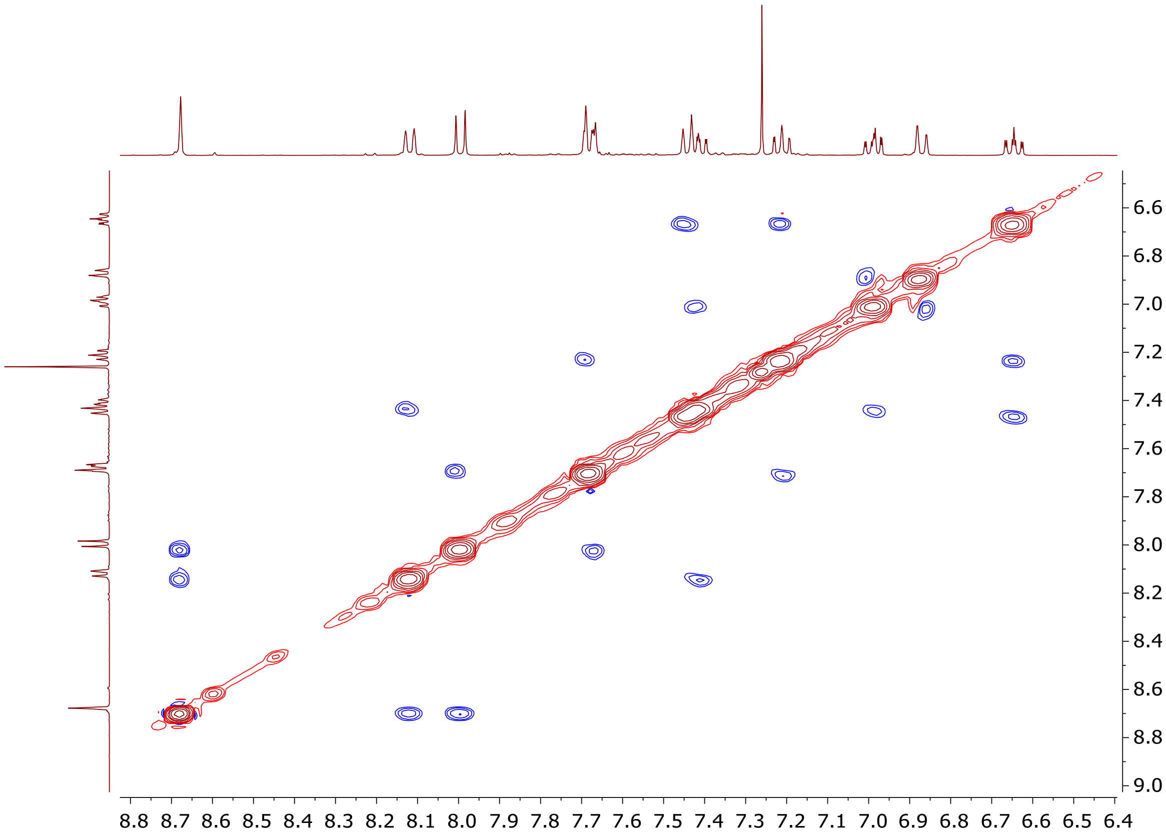

$^{13}C\{^{1}H\}$ NMR (101 MHz, $CDCl_3$) spectra of (*aR*)-**5**:

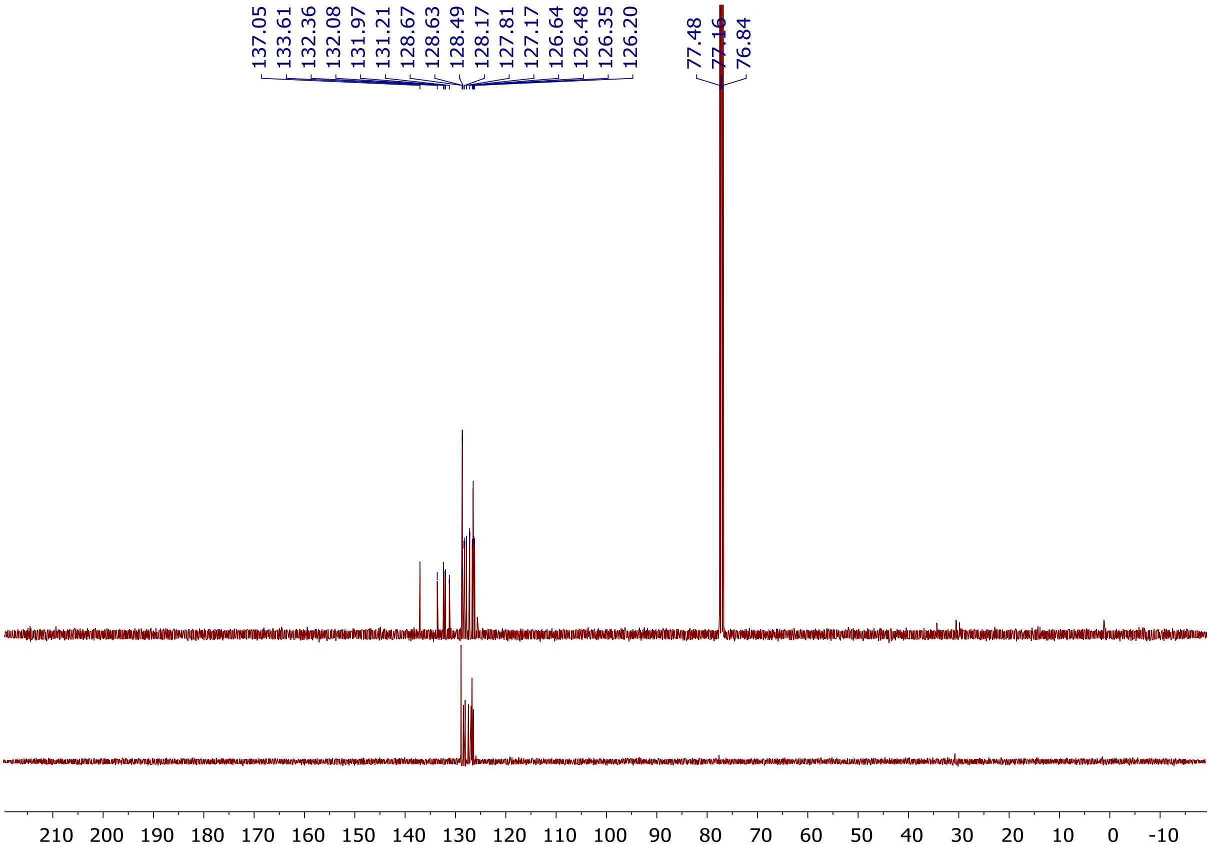

HPLC chromatogram of racemic **5** (Chiralpak IB N-5 column, heptane/dichloromethane = 95:5, 1 mL/min):

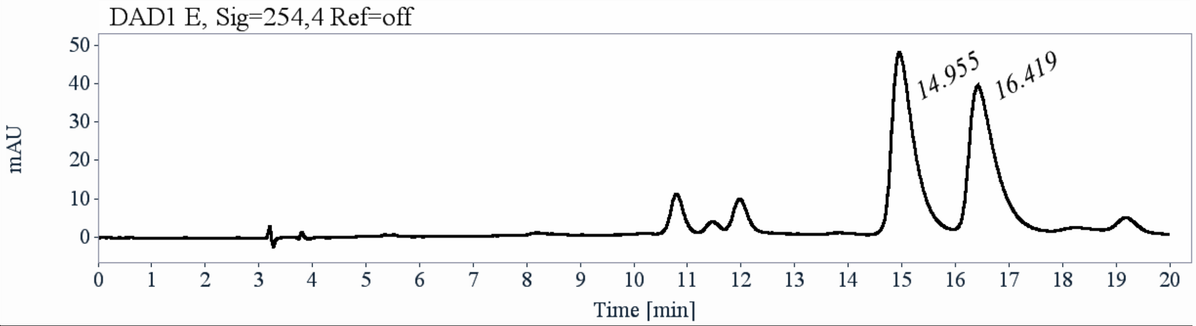

RT [min]	Area	Area%	Capacity Factor	Enantioselectivity	Resolution (USP)
14.95	1384	51.46	4.07		
16.42	1306	48.54	4.57	1.12	1.84
Sum	2690	100.00			

HPLC chromatogram of (*aR*)-**5** (Chiralpak IB N-5 column, heptane/dichloromethane = 95:5, 1 mL/min):

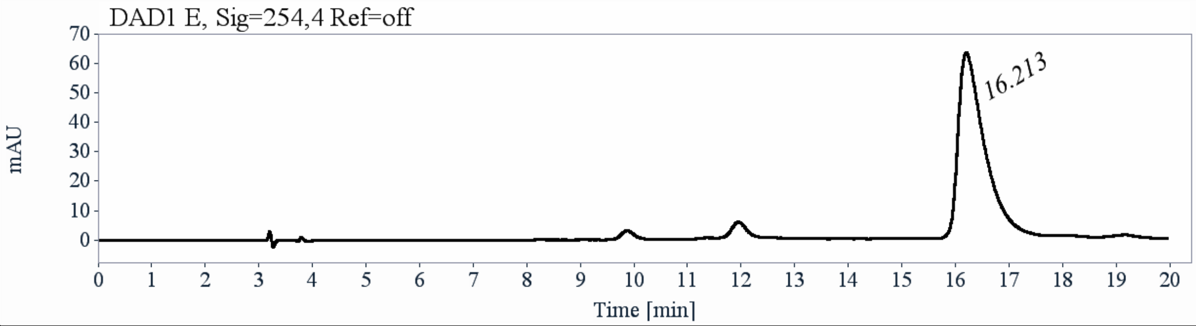

RT [min]	Area	Area%	Capacity Factor	Enantioselectivity	Resolution (USP)
16.21	2277	100.00	4.50		
Sum	2277	100.00			

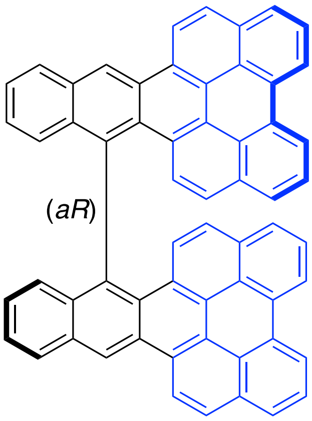

(*aR*)-**6**
Chemical Formula: $C_{60}H_{30}$
Molecular Weight: 750,90

Following the general procedure 1 with (*aR*)-**1** (100 mg, 0.12 mmol, 99% *ee*) and perylene (63 mg, 0.25 mmol) in 5 mL of Et_2O after 16 hours afforded the crude product. Purification by flash column chromatography eluted with pentane/EtOAc = 10:1 afforded (*aR*)-**6** (4 mg, 4%) as a red solid. **Mp** >360 °C (amorphous); **TLC** (pentane/EtOAc = 10:1) R*f* = 0.35; **HRMS** (ESI+) *m/z* calcd for $C_{60}H_{30}Ag^+$ $[M+Ag]^+$ 859.1403, found 859.1403; **^{1}H NMR** (400 MHz, $CDCl_3$) δ 9.96 (s, 2H), 9.38 (d, *J* = 9.2 Hz, 2H), 8.77 (d, *J* = 8.2 Hz, 2H), 8.69 (d, *J* = 7.3 Hz, 2H), 8.61 (d, *J* = 9.4 Hz, 2H), 8.34 (dd, *J* = 9.4, 8.5 Hz, 4H), 8.17 (d, *J* = 7.6 Hz, 2H), 7.91 (dd, *J* = 8.3, 7.7 Hz, 2H), 7.70–7.63 (m, 4H), 7.43 (ddd, *J* = 8.7, 6.6, 1.1 Hz, 2H), 7.14 (d, *J* = 9.6 Hz, 2H), 6.88 (dd, *J* = 8.3, 1.2 Hz, 1H), 6.86 (dd, *J* = 8.3, 1.2 Hz, 1H), 6.75 (dd, *J* = 9.4, 0.9 Hz, 2H); it was not possible to perform a conclusive ^{13}C NMR analysis because of the too small amount of material available; **HPLC** Chiralpak IA column, heptane/dichloromethane = 80:20, 1 mL/min, 310 nm, retention time: t_{major} = 12.87 min, t_{minor} = 14.58 min, 98% *ee*; **Specific rotation** $[\alpha]_D^{25}$ = -398.2 (c = 1.0, $CHCl_3$).

^{1}H NMR (400 MHz, $CDCl_3$) spectrum of (*aR*)-**6**:

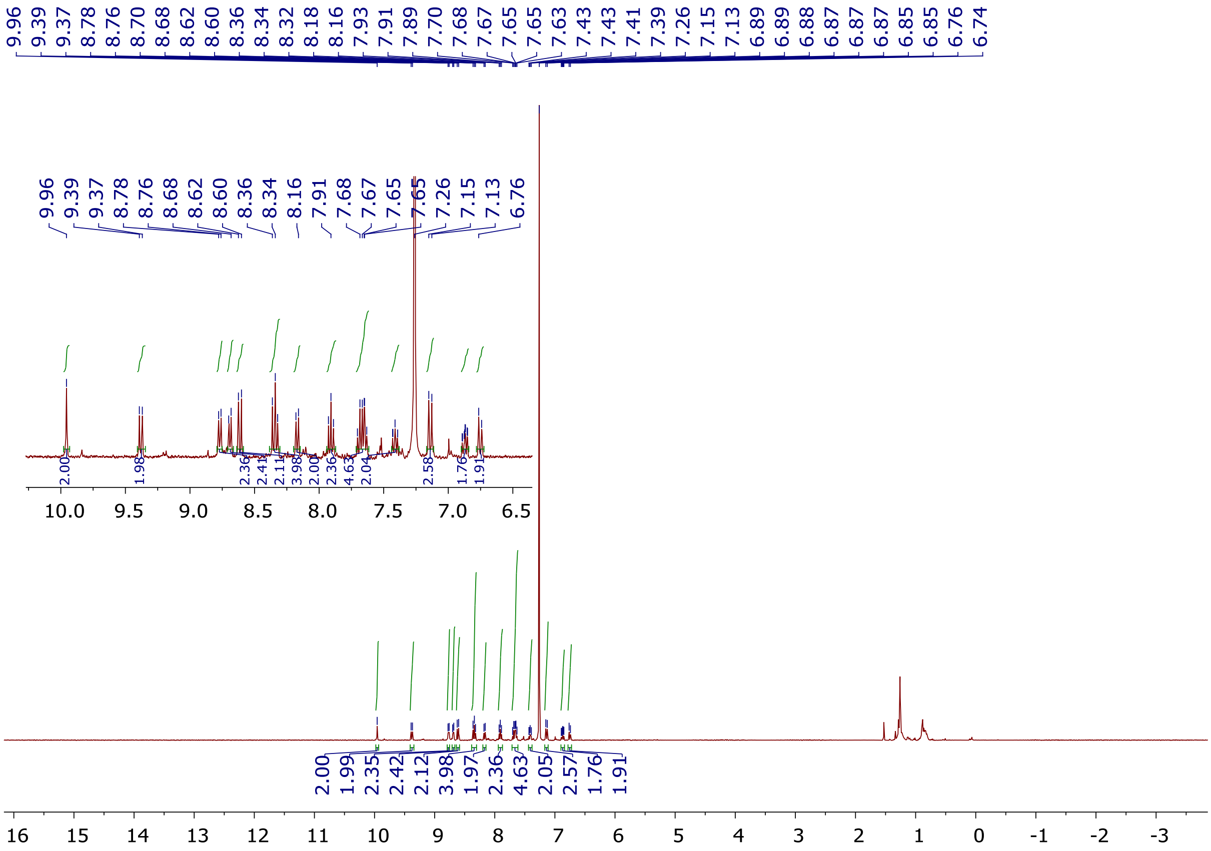

HPLC chromatogram of racemic **6** (Chiralpak IA column, heptane/dichloromethane = 80:20, 1 mL/min):

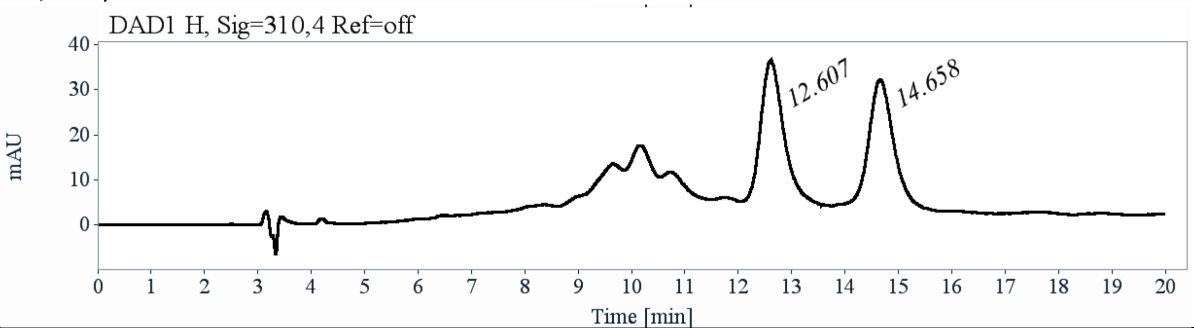

RT [min]	Area	Area%	Capacity Factor	Enantioselectivity	Resolution (USP)
12.61	948	50.24	3.27		
14.66	939	49.76	3.97	1.21	2.59
Sum	1887	100.00			

HPLC chromatogram of (*aR*)-**6** (Chiralpak IA column, heptane/dichloromethane = 80:20, 1 mL/min):

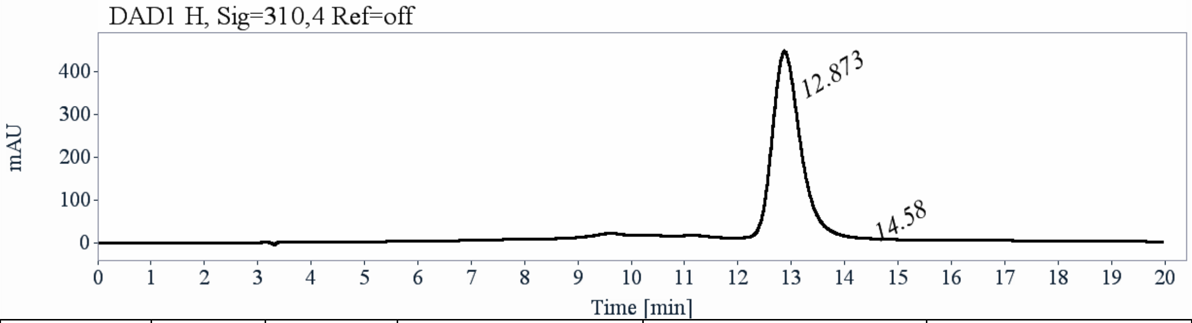

RT [min]	Area	Area%	Capacity Factor	Enantioselectivity	Resolution (USP)
12.87	15704	98.82	3.36		
14.58	188	1.18	3.94	1.17	
Sum	15892	100.00			

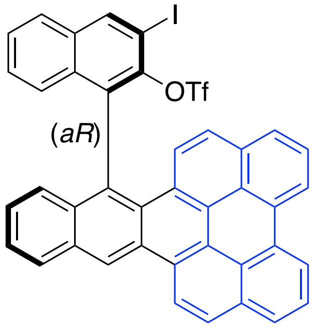

(*aR*)-**7**

Chemical Formula: $C_{41}H_{20}F_3IO_3S$
Molecular Weight: 776,57

Following the general procedure 1 with (*aR*)-**1** (100 mg, 0.12 mmol, 99% *ee*) and perylene (94 mg, 0.37 mmol) in 5 mL of Et_2O after 2 hours afforded the crude product. Purification by flash column chromatography eluted with pentane/Et_2O = 10:1 afforded (*aR*)-**7** (20 mg, 21%) as an orange solid. **Mp** 262–264 °C (amorphous); **TLC** (pentane/EtOAc = 10:1) R*f* = 0.59; **HRMS** (ESI+) *m/z* calcd for $C_{41}H_{21}F_3IO_3S^+$ [M+H]$^+$ 777.0203, found 777.0206; **^{1}H NMR** (400 MHz, $CDCl_3$) δ 9.85 (s, 1H), 9.19 (d, *J* = 9.5 Hz, 1H), 8.83 (s, 1H), 8.74 (d, *J* = 7.7 Hz, 1H), 8.71 (d, *J* = 7.3 Hz, 1H), 8.38 (d, *J* = 8.4 Hz, 1H), 8.24 (d, *J* = 8.4 Hz, 1H), 8.09 (d, *J* = 8.4 Hz, 1H), 7.99 (d, *J* = 8.4 Hz, 1H), 7.86 (dd, *J* = 8.3, 7.7 Hz, 1H), 7.82 (dd, *J* = 9.5, 7.4 Hz, 1H), 7.78 (dd, *J* = 8.6, 7.8 Hz, 1H), 7.66 (ddd, *J* = 9.3, 6.8, 0.8 Hz, 1H), 7.58 (ddd, *J* = 9.3, 7.0, 1.0 Hz, 1H), 7.55 (d, *J* = 9.4 Hz, 2H), 7.47–7.43 (m,1H), 7.40 (s,1H), 7.36 (d, *J* = 9.5 Hz, 1H), 7.35 (d, *J* = 8.5Hz, 1H), 7.24 (dd, *J* = 7.8,1.0 Hz, 1H); **^{13}C{^{1}H} NMR** (101 MHz, $CDCl_3$) δ 145.4 (C), 141.9 (CH), 135.3 (C), 134.2 (C), 133.9 (C), 133.1 (C), 132.6 (C), 131.9 (C), 131.3 (C), 131.3 (C), 131.2 (C), 131.1 (C), 128.8 (CH), 128.7 (CH), 128.5 (CH) 128.4 (CH), 128.4 (C), 128.2 (C), 128.0 (CH), 127.4 (CH), 127.4 (C), 127.4 (CH), 127.1 (CH), 127.0 (CH), 126.9 (3CH), 126.9 (C), 126.8 (C), 126.6 (C), 126.4 (CH), 126.4 (C), 126.3 (C), 126.1 (CH), 126.1 (CH), 126.0 (CH), 125.2 (CH), 120.9 (CH), 120.6 (CH), 117.9 (q, $^1J_{C-}$

$_F$ = 322.6 Hz, CF_3), 87.6 (C); **^{19}F NMR** (376 MHz, $CDCl_3$) δ -77.5; **HPLC** Chiralpak IB N-5 column, heptane/ethanol = 90:10, 1 mL/min, 254 nm, retention time: t_{minor} = 9.81 min, t_{major} = 10.88 min, 99% *ee*; **Specific rotation** $[\alpha]_D^{25}$ = -214.5 (c = 1.0, $CHCl_3$).

^{1}H NMR (400 MHz, $CDCl_3$) spectrum of (*aR*)-**7**:

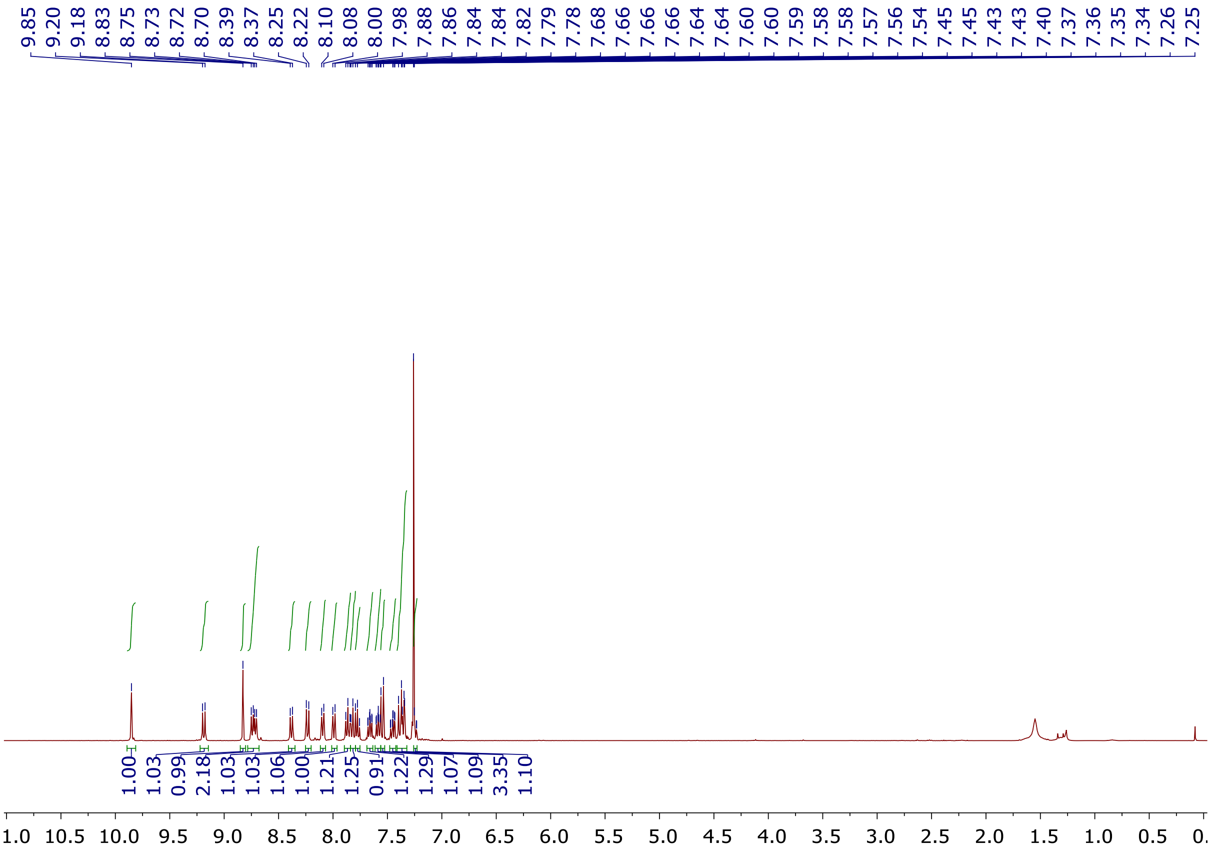

$^{13}C\{^1H\}$ NMR (101 MHz, $CDCl_3$) spectra of (*aR*)-**7**:

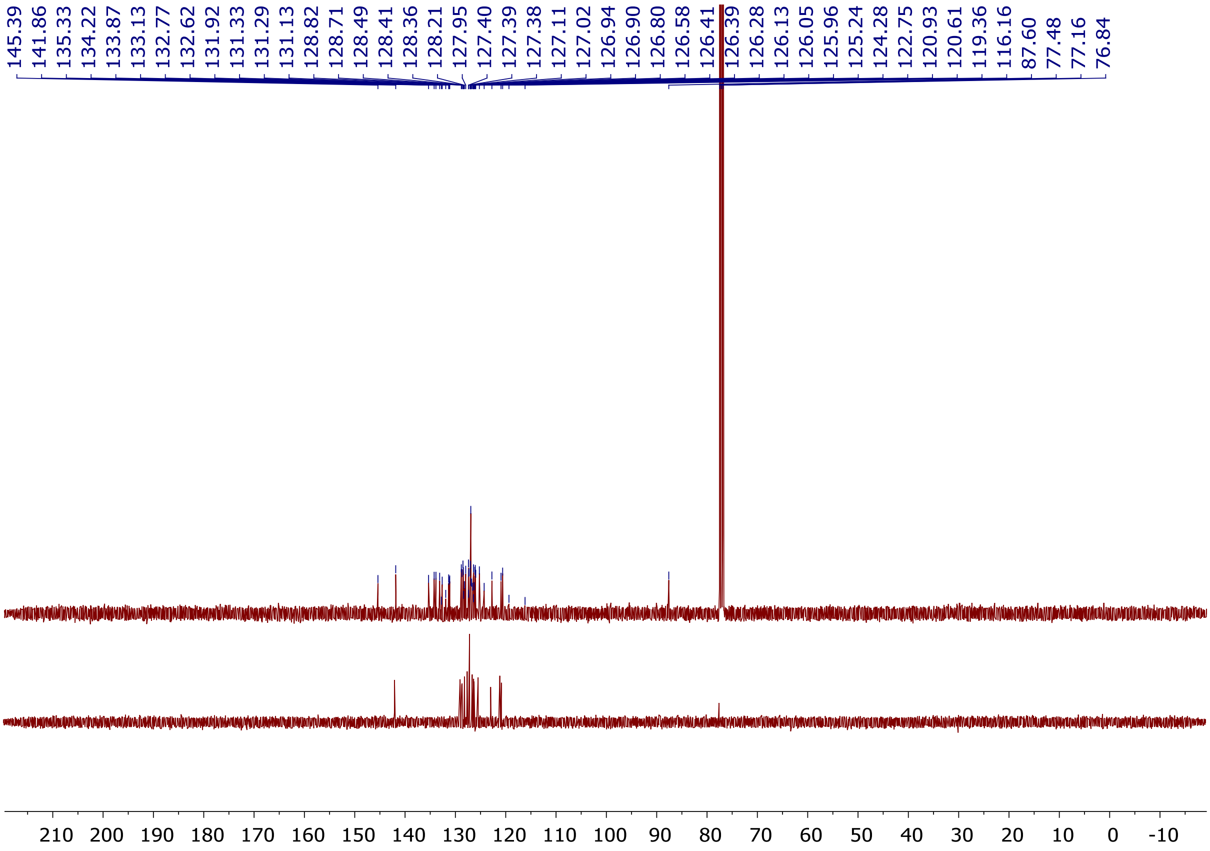

HPLC chromatogram of racemic **7** (Chiralpak IB N-5 column, heptane/ethanol = 90:10, 1 mL/min):

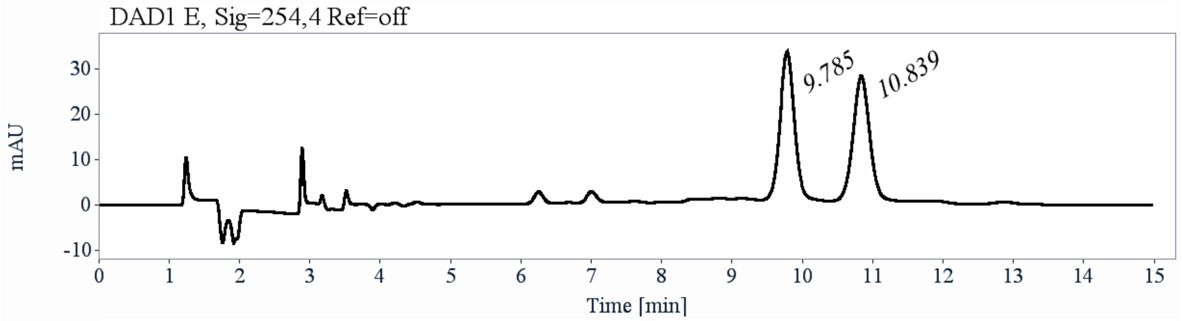

RT [min]	Area	Area%	Capacity Factor	Enantioselectivity	Resolution (USP)
9.79	468	50.85	2.32		
10.84	453	49.15	2.67	1.15	2.64
Sum	921	100.00			

HPLC chromatogram of (*aR*)-**7** (Chiralpak IB N-5 column, heptane/ethanol = 90:10, 1 mL/min):

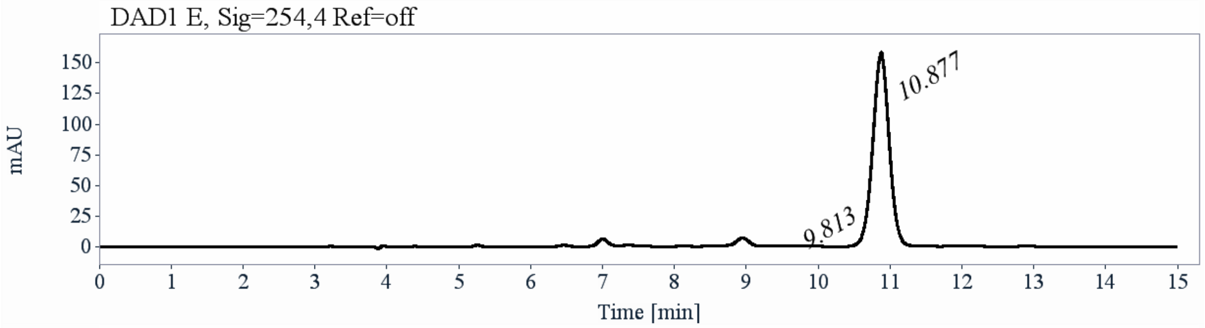

RT [min]	Area	Area%	Capacity Factor	Enantioselectivity	Resolution (USP)
9.81	9	0.37	2.33		
10.88	2500	99.63	2.69	1.15	2.50
Sum	2510	100.00			

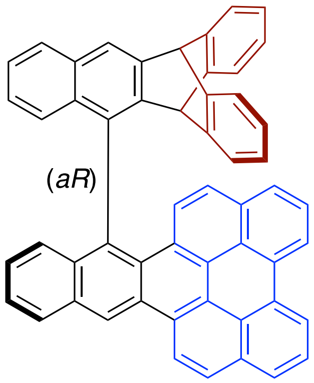

(*aR*)-**8**
Chemical Formula: $C_{54}H_{30}$
Molecular Weight: 678,83

A round bottom flask containing the aryne precursor (*aR*)-**7** (20 mg, 0.025 mmol, 99% *ee*) was charged with 2 mL of Et_2O and anthracene (18 mg, 0.10 mmol) under an argon atmosphere. The suspension was cooled down to 0 °C and trimethylsilylmethylmagnesium chloride (1.0 M in Et_2O, 0.50 mL, 0.50 mmol) was added at this temperature at once. After 16 h at 0 °C, the reaction mixture was hydrolyzed with water. The mixture was extracted three times with EtOAc and the combined organic layers were washed with brine, dried over anhydrous Na_2SO_4, filtered, and concentrated under reduced pressure. The residue was purified by flash chromatography eluted with pentane/EtOAc = 10:1 to afford (*aR*)-**8** (4 mg, 28%) as an orange solid. **Mp** 286–288 °C (amorphous); **TLC** (pentane/EtOAc = 10:1) R*f* = 0.32; **HRMS** (ESI+) *m/z* calcd for $C_{54}H_{30}Ag^+$ $[M+H]^+$ 787.1401, found 787.1396; ^{1}**H NMR** (500 MHz, $CDCl_3$) δ 9.89 (s, 1H), 9.31 (d, *J* = 9.0 Hz, 1H), 8.78 (d, *J* = 7.5 Hz, 1H), 8.70 (d, *J* = 7.5 Hz, 1H), 8.44 (d, *J* = 8.4 Hz, 1H), 8.31 (d, *J* = 9.0 Hz, 1H), 8.15 (d, *J* = 7.5 Hz, 1H), 8.06 (s, 1H), 7.90 (dd, *J* = 9.0, 7.5 Hz, 2H), 7.71 (dd, *J* = 8.3, 7.7 Hz, 1H), 7.64 (ddd, *J* = 8.2, 6.3, 1.0 Hz, 1H), 7.54 (d, *J* = 7.4 Hz, 1H), 7.44 (d, *J* = 8.4 Hz, 1H), 7.38–7.36 (m, 2H), 7.32 (d, *J* = 9.5 Hz, 1H), 7.22–7.14 (m, 3H), 7.07 (ddd, *J* = 8.5, 6.9, 0.9 Hz, 1H), 6.96 (dd, *J* = 8.8, 8.0 Hz, 1H), 6.77–6.71 (m, 2H), 6.63 (d, *J* = 9.4 Hz, 1H),

6.49 (d, J = 6.7 Hz, 1H), 5.90 (ddd, J = 8.2, 7.3, 0.7 Hz, 1H), 5.80 (d, J = 7.2 Hz, 2H), 5.64 (s, 1H), 4.55 (s, 1H); **^{13}C{^{1}H} NMR** (126 MHz, $CDCl_3$) δ 144.8 (C), 144.2 (C), 144.0 (C), 143.0 (C), 142.9 (C), 141.0 (C), 134.5 (C), 133.2 (C), 132.8 (C), 132.6 (C), 132.3 (C), 132.0 (C), 131.7 (C), 131.4 (C), 131.4 (C), 130.9 (C), 130.2 (CH), 129.9 (CH), 128.5 (CH), 128.2 (CH), 128.0 (CH), 127.7 (C), 127.5 (CH), 127.3 (C), 127.2 (CH), 127.1 (C), 126.9 (2CH), 126.8 (CH), 126.7 (C), 126.6 (CH), 126.5 (CH), 126.4 (CH), 126.1 (CH), 125.6 (C), 125.5 (CH), 125.4 (CH), 125.3 (CH), 124.9 (CH), 124.8 (CH), 124.6 (C), 124.5 (C), 124.3 (CH), 124.0 (CH), 123.5 (CH), 123.4 (CH), 123.0 (CH), 122.9 (CH), 122.4 (C), 121.8 (CH), 120.8 (CH), 120.2 (CH), 54.4 (CH), 51.4 (CH); **HPLC** Chiralpak IB N-5 column, heptane/dichloromethane = 80:20, 1 mL/min, 310 nm, retention time: t_{major} = 8.50 min, t_{minor} = 9.15 min, 99% *ee*; **Specific rotation** $[\alpha]_D^{25}$ = -198.3 (c = 1.0, $CHCl_3$).

^{1}H NMR (400 MHz, $CDCl_3$) spectrum of (*aR*)-**8**:

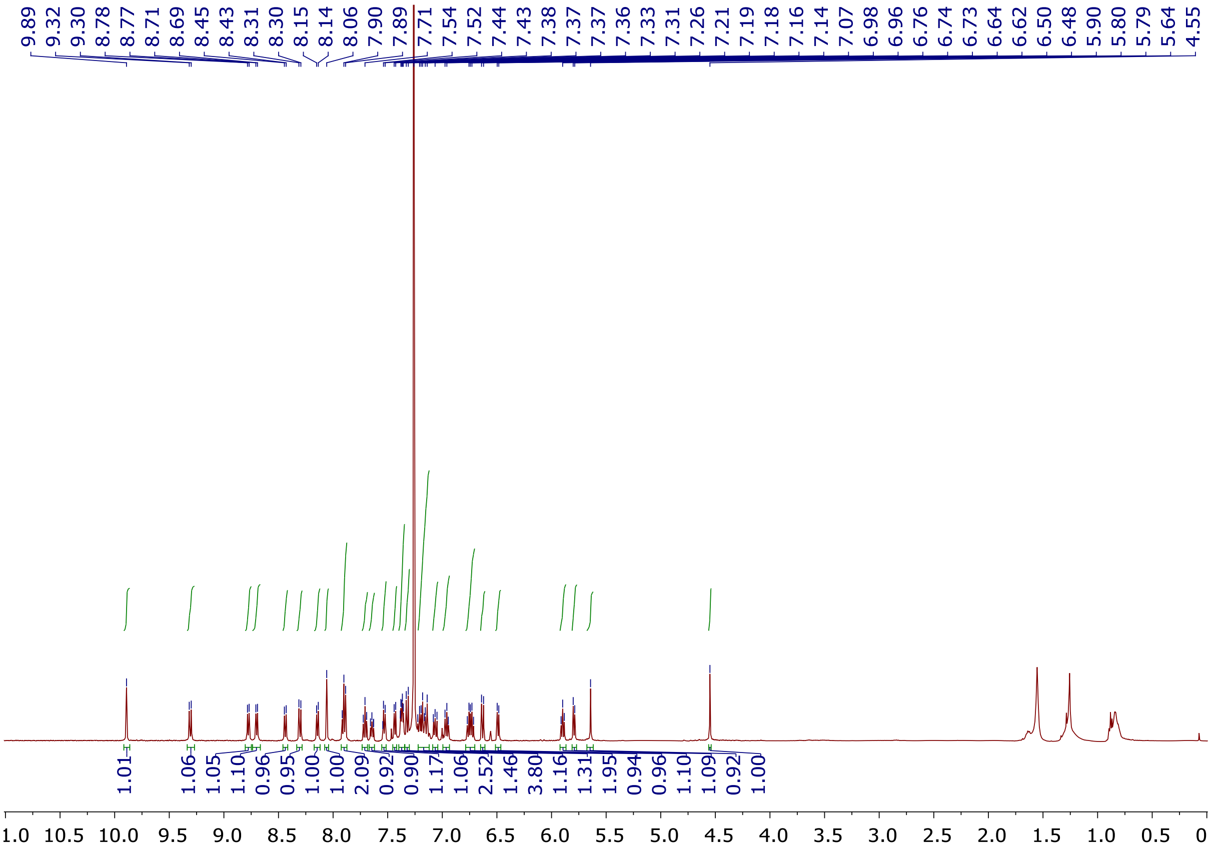

$^{13}C\{^1H\}$ NMR (126 MHz, $CDCl_3$) spectra of (*aR*)-**8**:

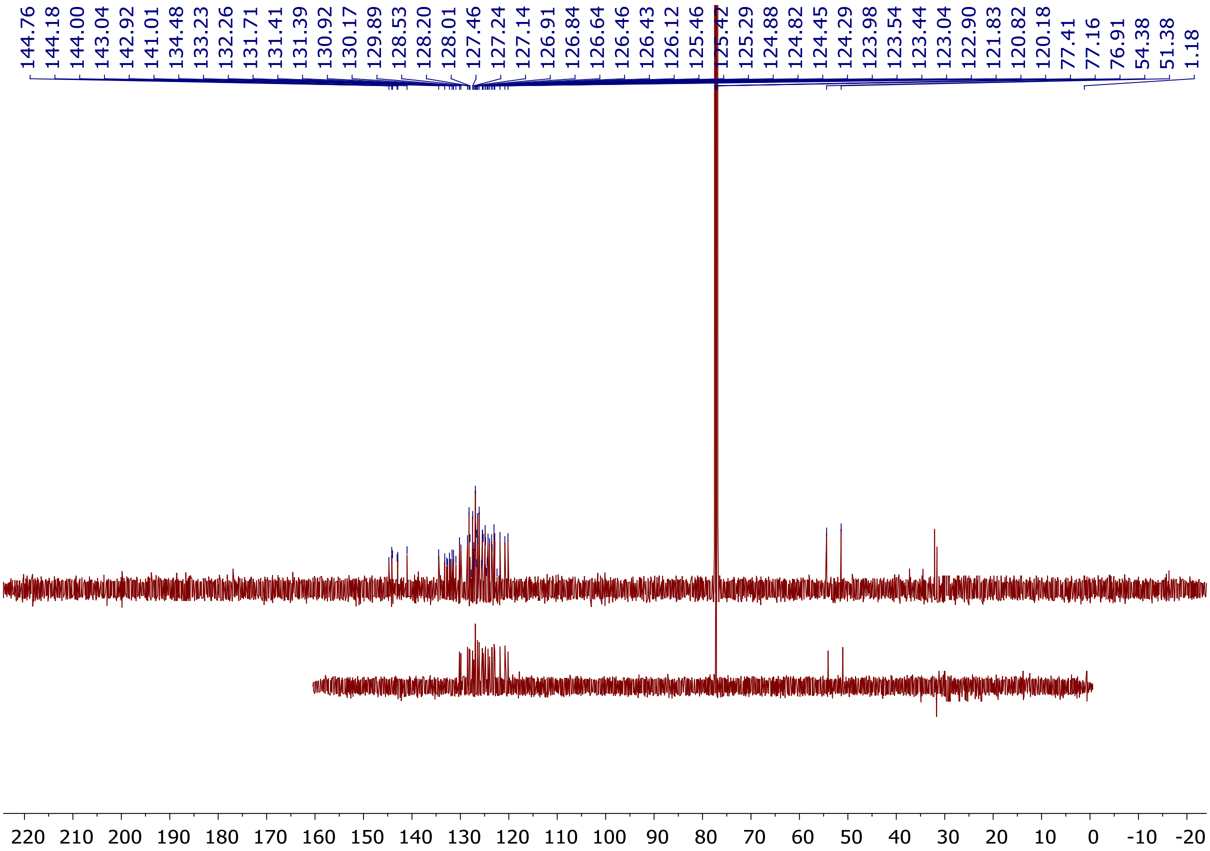

HPLC chromatogram of racemic **8** (Chiralpak IB N-5 column, heptane/dichloromethane = 80:20, 1 mL/min):

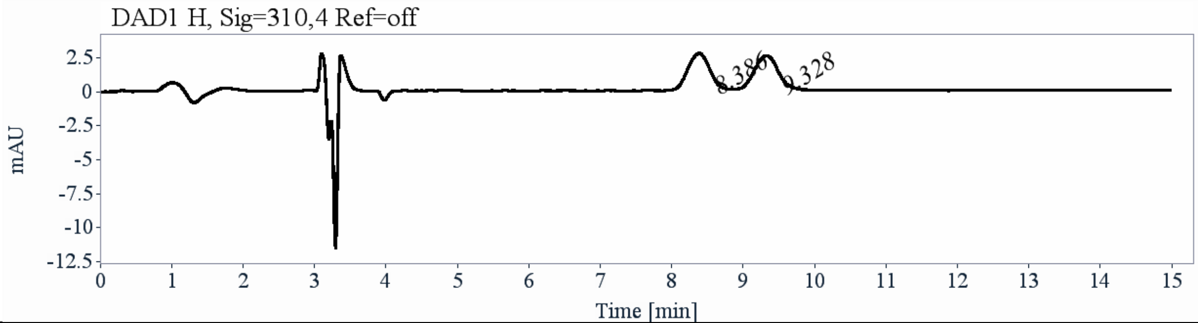

RT [min]	Area	Area%	Capacity Factor	Enantioselectivity	Resolution (USP)
8.39	57	49.40	1.84		
9.33	59	50.60	2.16	1.17	1.65
Sum	116	100.00			

HPLC chromatogram of (*aR*)-**8** (Chiralpak IB N-5 column, heptane/dichloromethane = 80:20, 1 mL/min):

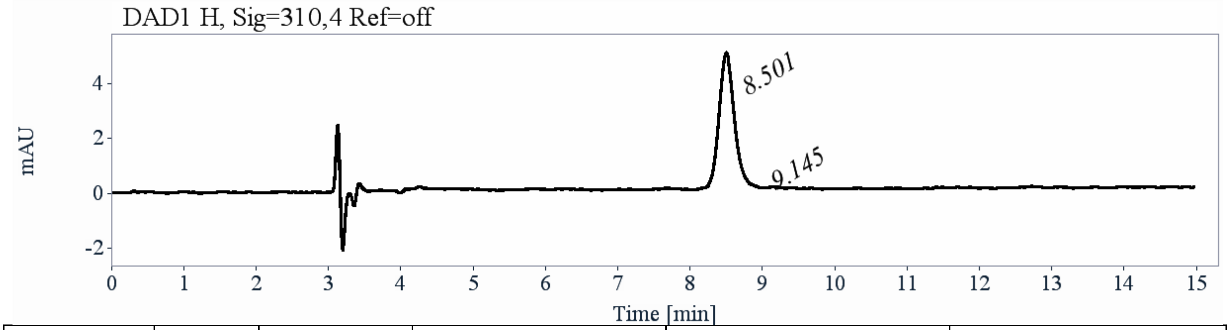

RT [min]	Area	Area%	Capacity Factor	Enantioselectivity	Resolution (USP)
8.50	74	99.46	1.88		
9.15	0	0.54	2.10	1.12	2.10
Sum	74	100.00			

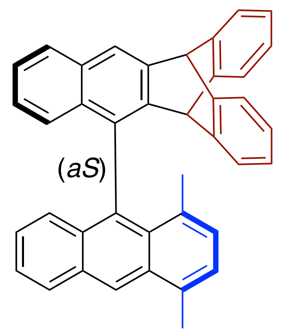

(*aS*)-**9**
Chemical Formula: $C_{40}H_{28}$
Molecular Weight: 508,66

In a round bottom flask, the aryne precursor (*aS*)-**1** (100 mg, 0.12 mmol, 99% *ee*) was solubilized with 3 mL anhydrous toluene, the toluene was evaporated in vacuo, and the flask was placed under an argon atmosphere (drying step). Then, the flask was charged with 5 mL of Et_2O and 2,5-dimethylfuran (20 µL, 0.19 mmol) under an argon atmosphere. The suspension was cooled down to 0 °C and trimethylsilylmethylmagnesium chloride (1.0 M in Et_2O, 2.50 mL, 2.50 mmol) was added at this temperature over 4 hours. The reaction was monitored by TLC and after stirring for 2 additional hours at 0 °C, anthracene (110 mg, 0.50 mmol) and trimethylsilylmethylmagnesium chloride (1.0 M in Et_2O, 2.50 mL, 2.50 mmol) were subsequently added at this temperature. After stirring 16 h at 0 °C, the mixture was hydrolyzed with water, extracted three times with EtOAc and the combined organic layers were washed with brine, dried over anhydrous Na_2SO_4, filtered, and concentrated under reduced pressure to afford the intermediate cycloadduct (1:1 mixture of diastereomers) as a green solid (23 mg). This material was directly reacted with NaI/Me_3SiCl according to the general procedure 2. Purification by flash chromatography eluted with pentane/Et_2O = 10:1 afforded (*aS*)-**9** (11 mg, 17%) as a white solid. **Mp** 193–195 °C (amorphous); **TLC** (pentane/Et_2O = 10:1) R*f* = 0.75; **HRMS** (ESI+) *m/z* calcd for $C_{40}H_{28}Ag^+$ $[M+Ag]^+$ 617.1240,

found 617.1239; **^{1}H NMR** (500 MHz, $CDCl_3$) δ 8.86 (s, 1H), 8.18 (d, *J* = 8.6 Hz, 1H), 7.95 (s, 1H), 7.80 (d, *J* = 8.6 Hz, 1H), 7.47–7.44 (m, 3H), 7.32 (ddd, *J* = 8.6, 6.9, 1.0 Hz, 1H), 7.25 (d, *J* = 7.3 Hz, 1H), 7.06 (ddd, *J* = 8.7, 6.9, 1.1 Hz, 1H), 7.08–6.98 (m, 3H), 6.94 (d, *J* = 7.0 Hz, 1H), 6.88–6.81 (m, 4H), 6.79 (d, *J* = 7.3 Hz, 1H), 6.56 (d, *J* = 7.3 Hz, 1H), 5.66 (s, 1H), 4.71 (s, 1H), 2.97 (s, 3H), 1.16 (s, 3H); **^{13}C{^{1}H} NMR** (126 MHz, $CDCl_3$) δ 145.0 (C), 145.0 (C), 144.2 (C), 143.5 (C), 142.0 (C), 141.2 (C), 134.1 (C), 134.1 (C), 133.2 (C), 132.8 (C), 132.6 (C), 132.5 (C), 131.6 (C), 131.5 (C), 131.3 (C), 130.8 (C), 129.1 (CH), 128.4 (CH), 127.7 (CH), 127.3 (CH), 126.7 (CH), 126.1 (CH), 125.8 (CH), 125.8 (CH), 125.7 (CH), 125.6 (CH), 125.5 (CH), 125.5 (CH), 125.3 (CH), 125.2 (CH), 124.9 (CH), 124.5 (CH), 124.2 (CH), 123.6 (CH), 123.5 (CH), 121.6 (CH), 54.3 (CH), 51.6 (CH), 23.9 (CH_3), 20.8 (CH_3); **HPLC** Chiralpak IJ column, heptane/dichloromethane = 85:15, 1 mL/min, 254 nm, retention time: t_{minor} = 4.23 min, t_{major} = 4.56 min, 99% *ee*; **Specific rotation** $[\alpha]_D^{25}$ = +148.1 (c = 1.0, $CHCl_3$).

^{1}H NMR (400 MHz, $CDCl_3$) spectrum of (*aS*)-**9**:

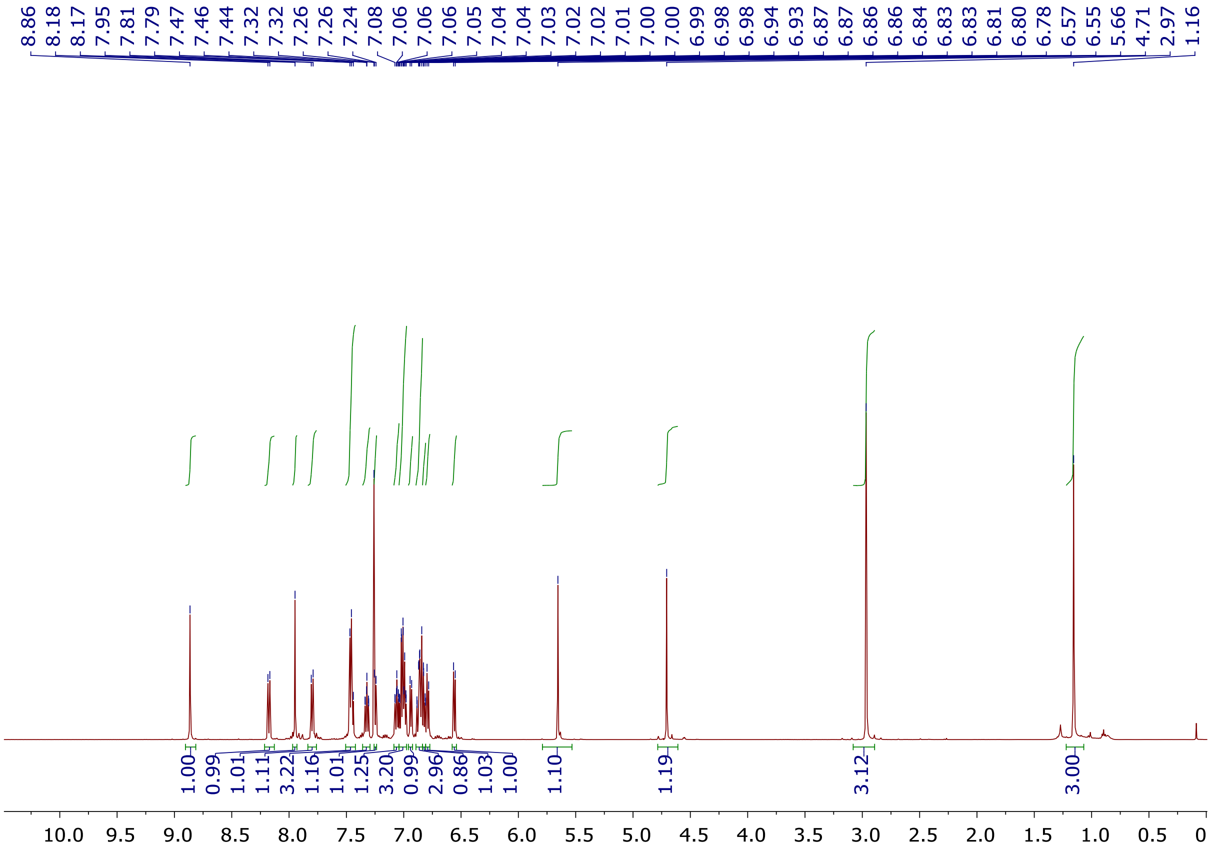

$^{13}C\{^{1}H\}$ NMR (126 MHz, $CDCl_3$) spectra of (*aS*)-**9**:

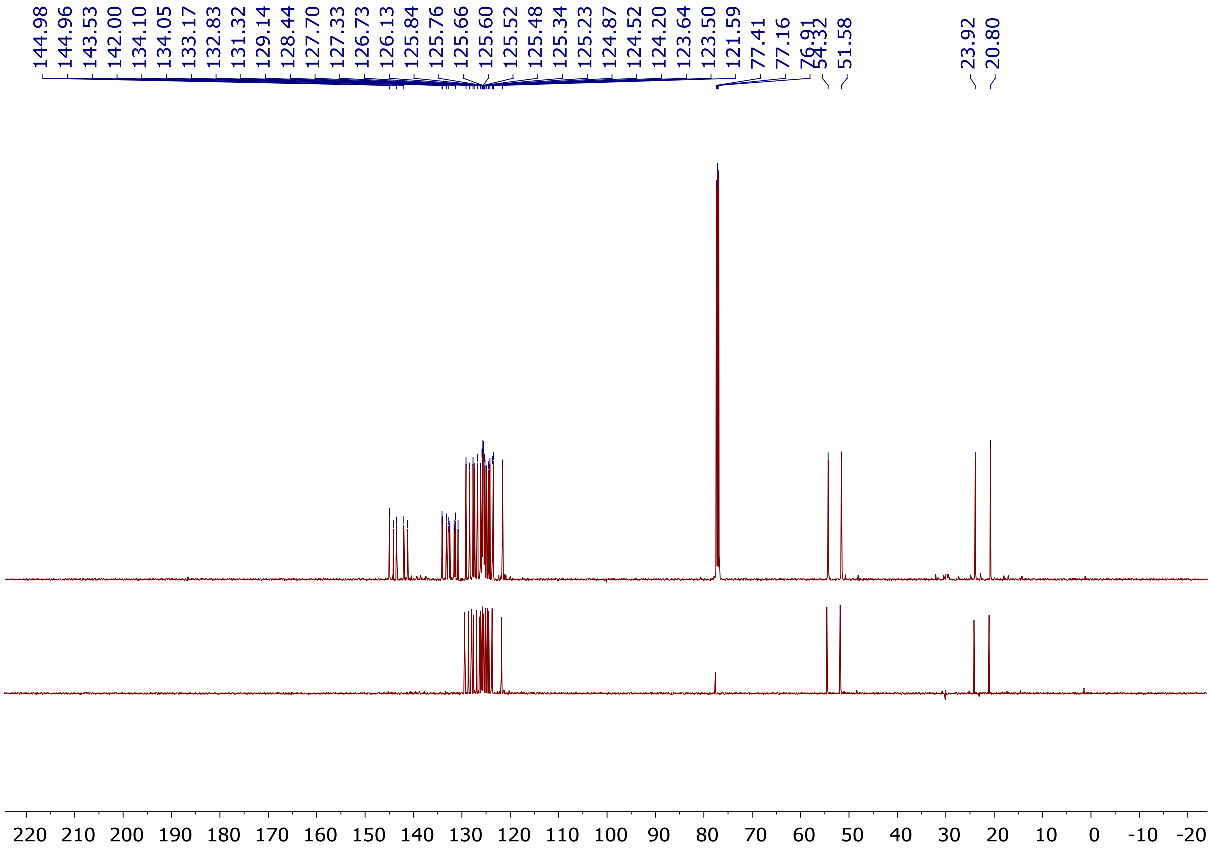

HPLC chromatogram of racemic **9** (Chiralpak IJ column, heptane/dichloromethane = 85:15, 1 mL/min):

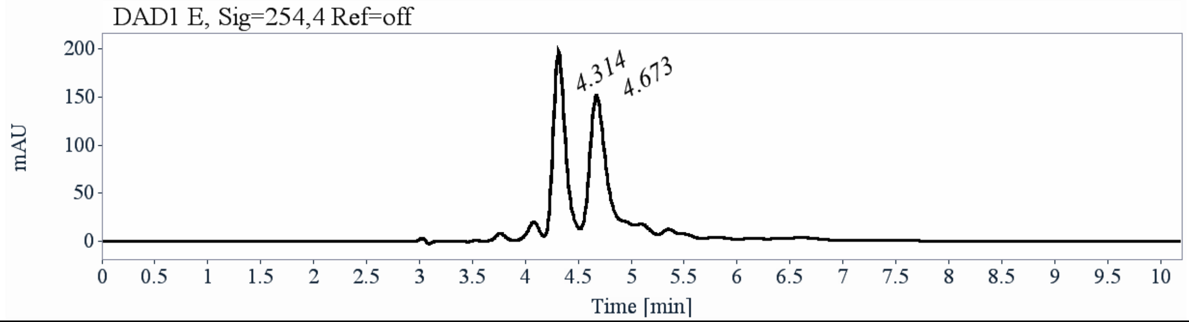

RT [min]	Area	Area%	Capacity Factor	Enantioselectivity	Resolution (USP)
4.31	1501	48.67	0.46		
4.67	1582	51.33	0.58	1.26	1.52
Sum	3083	100.00			

HPLC chromatogram of (*aS*)-**9** (Chiralpak IJ column, heptane/dichloromethane = 85:15, 1 mL/min):

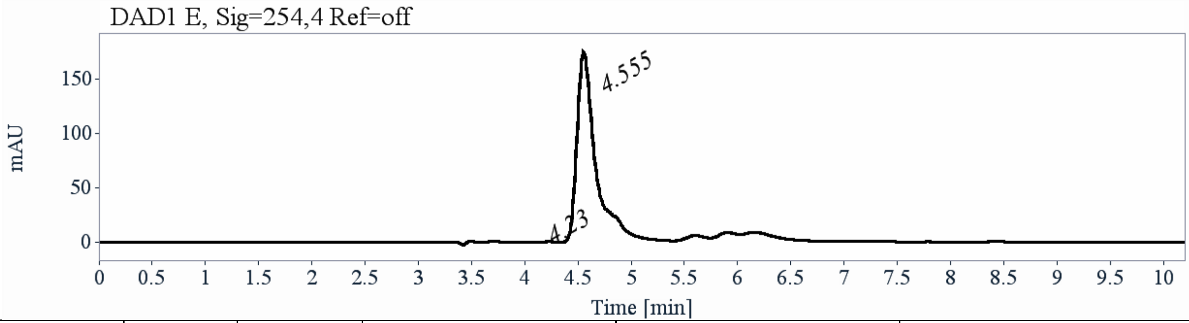

RT [min]	Area	Area%	Capacity Factor	Enantioselectivity	Resolution (USP)
4.23	4	0.17	0.43		
4.56	2107	99.83	0.54	1.25	1.43
Sum	2111	100.00			

2. Computational studies

The density functional theory (DFT) calculations reported in this section were performed with the Gaussian16 suite.[9] All geometries were fully optimized using either B3LYP,[10] M062X, [11] or BP86[12] density functional with either 6-31G(d), 6-311++G(d,p),[13] cc-pVDZ,[14] aug-cc-pVDZ, [14] or cc-pVTZ,[14] basis set with pseudo potential correction[15] for I atoms in the gas phase or using the IEFPCM solvation model[16] or its SMD variation[17] for diethyl ether. Dispersion effects were accounted for using Grimme's D3 dispersion model.[18] For all stationary points, the second derivatives were analytically calculated in order to determine if a minimum (zero negative eigenvalue) or a transition state (one negative eigenvalue) existed for this geometry. The connection between the transition states and the corresponding minima was performed manually by steepest descend optimization. All energies are relative Gibbs free energies expressed in kJ·mol^{-1} as computed at 298 K unless otherwise stated.

*Configurational stability of the elusive bis(aryne) atropisomer derived from precursor **1***

Exploration of the potential energy surface for the conformations of the elusive bis(aryne) atropisomer derived from precursor **1** was first performed at the BP86/cc-pVDZ level of theory in the gas phase. Two possible transitions states corresponding to the rotation around the stereogenic axis could be located, with **TS-enant-bisaryne#1** having the H atoms of the naphthyl moieties overhanging the aryne triple bonds being favored by 59.8 kJ·mol^{-1} over **TS-enant-bisaryne#2** (Figure S5). Next, the geometries and Gibbs free energies of the kinetically favored enantiomerization pathway were recomputed at the B3LYP-D3/6-311++G(d,p) level of theory including a solvation model (IEFPCM) for diethyl ether. The enantiomerization barrier of 1,1'-binaphthalene was computed at the same level of theory for comparison.

---

[9] Gaussian 16, Revision A.03, M. J. Frisch, G. W. Trucks, H. B. Schlegel, G. E. Scuseria, M. A. Robb, J. R. Cheeseman, G. Scalmani, V. Barone, G. A. Petersson, H. Nakatsuji, X. Li, M. Caricato, A. V. Marenich, J. Bloino, B. G. Janesko, R. Gomperts, B. Mennucci, H. P. Hratchian, J. V. Ortiz, A. F. Izmaylov, J. L. Sonnenberg, D. Williams-Young, F. Ding, F. Lipparini, F. Egidi, J. Goings, B. Peng, A. Petrone, T. Henderson, D. Ranasinghe, V. G. Zakrzewski, J. Gao, N. Rega, G. Zheng, W. Liang, M. Hada, M. Ehara, K. Toyota, R. Fukuda, J. Hasegawa, M. Ishida, T. Nakajima, Y. Honda, O. Kitao, H. Nakai, T. Vreven, K. Throssell, J. A. Montgomery, Jr., J. E. Peralta, F. Ogliaro, M. J. Bearpark, J. J. Heyd, E. N. Brothers, K. N. Kudin, V. N. Staroverov, T. A. Keith, R. Kobayashi, J. Normand, K. Raghavachari, A. P. Rendell, J. C. Burant, S. S. Iyengar, J. Tomasi, M. Cossi, J. M. Millam, M. Klene, C. Adamo, R. Cammi, J. W. Ochterski, R. L. Martin, K. Morokuma, O. Farkas, J. B. Foresman, and D. J. Fox, Gaussian, Inc., Wallingford CT, 2016.

[10] (a) A. D. Becke, *Phys. Rev.* **1988**, *A38*, 3098–3100. (b) A. D. Becke, *J. Chem. Phys.* **1993**, *98*, 5648–5652. (c) C. Lee, W. Yang, R. G. Parr, *Phys. Rev.* **1988**, *B37*, 785–789.

[11] Y. Zhao, D. G. Truhlar, *Theor. Chem. Acc.* **2008**, *120*, 215–241.

[12] J. P. Perdew, *Phys. Rev. B* **1986**, *33*, 8822–8824.

[13] (a) W. J. Hehre, R. Ditchfield, J. A. Pople, *J. Chem. Phys.* **1972**, *56*, 2257–2261. (b) V. A. Rassolov, J. A. Pople, M. A. Ratner, T. L. Windus, *J. Chem. Phys.* **1998**, *109*, 1223–1229.

[14] (a) T. H. Dunning, Jr., *J. Chem. Phys.* **1989**, *90*, 1007–1023. (b) D. E. Woon, T. H. Dunning, Jr., *J. Chem. Phys.* **1993**, *98*, 1358–1371.

[15] M. Dolg, P. Fulde, H. Stoll, H. Preuss, A. Chang, R. M. Pitzer, *Chemical Physics* **1995**, *195*, 71–82.

[16] G. Scalmani, M. J. Frisch, *J. Chem. Phys.* **2010**, *132*, 114110.

[17] Marenich, A. V.; Cramer, C. J.; Truhlar, D. G. *J. Phys. Chem. B* **2009**, *113*, 6378–6396.

[18] S. Grimme, J. Antony, S. Ehrlich, H. Krieg, *J. Chem. Phys.* **2010**, *132*, 154104.

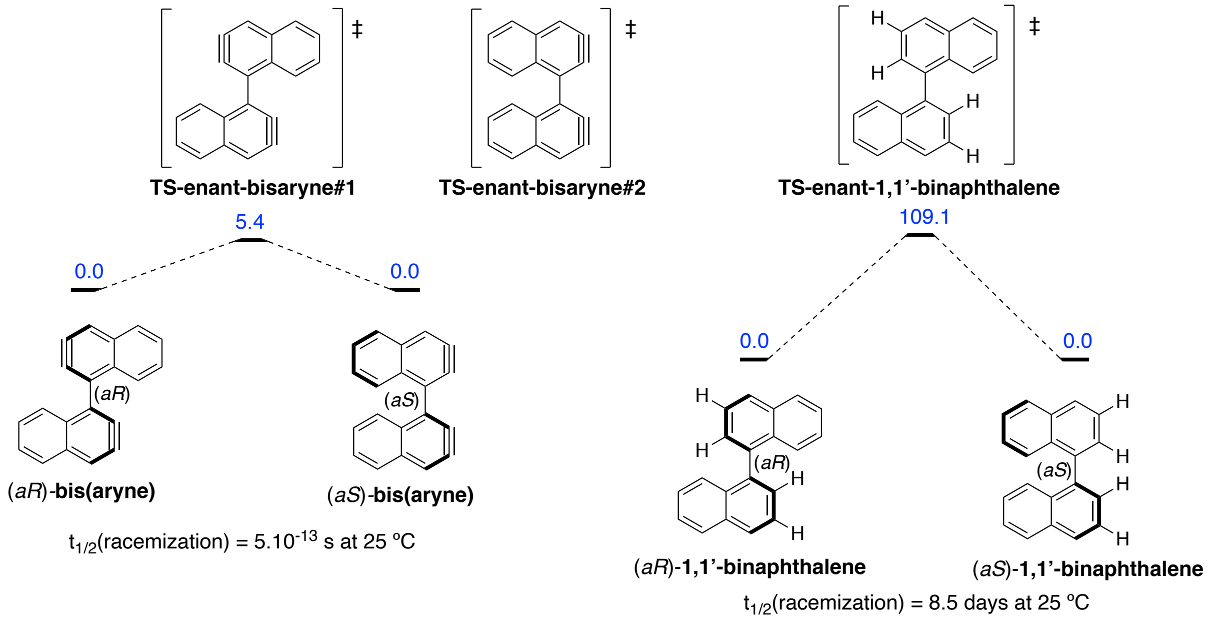

Figure S5. Enantiomerization of the elusive bis(aryne) atropisomer derived from precursor **1** and of 1,1'-binaphthalene. Relative Gibbs free energies expressed in kJ·mol^{-1} calculated at 298 K using B3LYP-D3/6-311++G(d,p) with the IEFPCM solvation model for diethyl ether.

*Configurational stability of the mono(aryne) atropisomer derived from precursor **1***

Exploration of the potential energy surface for the conformations of the aryne atropisomer derived from the mono reaction of precursor **1** with trimethylsilylmethylmagnesium chloride was first performed at the BP86/cc-pVDZ level of theory with pseudo potential correction for I atom in the gas phase. Two possible transitions states corresponding to the rotation around the stereogenic axis could be located, with **TS-enant-aryne#1** having the H atom of the naphthyl moiety overhanging the triflate group favored by 7.6 kJ·mol^{-1} over **TS-enant-aryne#2** (Figure S6). Next, the geometries and Gibbs free energies of the kinetically favored enantiomerization pathway were recomputed at the B3LYP-D3/cc-pVTZ level of theory with pseudo potential correction for I atom including the iefpcm solvation model for diethyl ether.

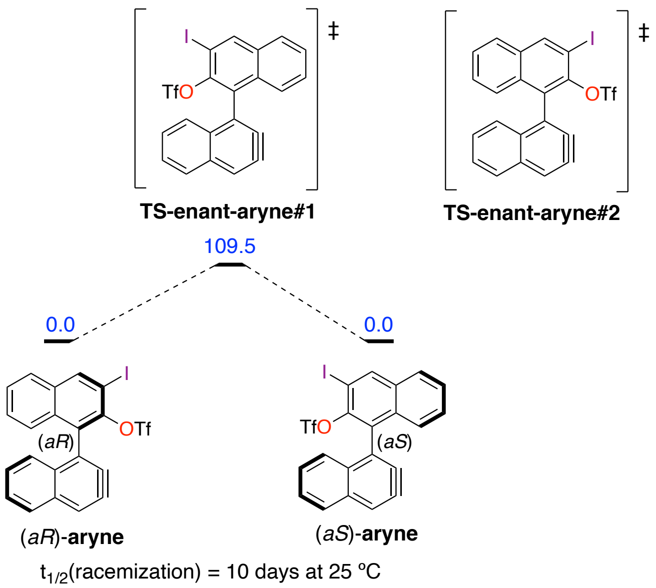

Figure S6. Enantiomerization of the aryne atropisomer derived from the mono reaction of precursor **1** with trimethylsilylmethylmagnesium chloride. Relative Gibbs free energies expressed in kJ·mol^{-1} calculated at 298 K using B3LYP-D3/cc-pVTZ with the iefpcm solvation model for diethyl ether.

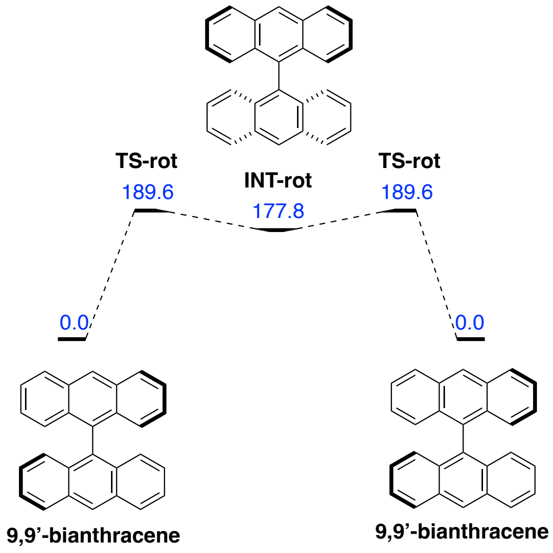

Figure S7. Rotation barrier around the C9–C9' single bond in 9,9'-bianthracene. Relative Gibbs free energies expressed in kJ·mol^{-1} calculated at 298 K M062X/aug-cc-pVDZ//M062X/cc-pVDZ using the SMD solvation model for diethyl ether.
